# Analyzing spatial distribution between ^18^F-fluorodeoxyglucose and ^18^F-boronophenylalanine positron emission tomography to investigate selection indicators for boron neutron capture therapy

**DOI:** 10.1186/s40658-022-00514-7

**Published:** 2022-12-19

**Authors:** Tetsu Nakaichi, Satoshi Nakamura, Kimiteru Ito, Kana Takahashi, Mihiro Takemori, Tairo Kashihara, Kouji Kunito, Naoya Murakami, Kotaro Iijima, Takahito Chiba, Hiroki Nakayama, Shohei Mikasa, Teiji Nishio, Hiroyuki Okamoto, Jun Itami, Hiroaki Kurihara, Hiroshi Igaki

**Affiliations:** 1 Radiation Safety and Quality Assurance Division, National Cancer Center Hospital, 5-1-1 Tsukiji, Chuo-ku, Tokyo 104-0045 Japan; 2 Division of Research and Development for Boron Neutron Capture Therapy, National Cancer Center Exploratory Oncology Research and Clinical Trial Center, 5-1-1 Tsukiji, Chuo-ku, Tokyo 104-0045 Japan; 3 Medical Physics Laboratory, Division of Health Science, Graduate School of Medicine, Osaka University, Yamadaoka 1-7, Suita City, Osaka 565-0871 Japan; 4 Department of Diagnostic Radiology, National Cancer Center Hospital, 5-1-1 Tsukiji, Chuo-ku, Tokyo 104-0045 Japan; 5 Department of Radiation Oncology, National Cancer Center Hospital, 5-1-1 Tsukiji, Chuo-ku, Tokyo 104-0045 Japan; 6 Department of Radiological Science, Graduate School of Human Health Science, Tokyo Metropolitan University, 7-2-10 Higashi-ogu, Arakawa-ku, Tokyo 116-8551 Japan; 7 Euro MediTech Co., Ltd., 2-20-4, Higashigotanda, Shinagawa-ku, Tokyo 141-0022 Japan; 8 Department of Diagnostic Radiology, Kanagawa Cancer Center, 2-3-2 Nakano, Asahi-ku, Yokohama, Kanagawa 241-8515 Japan

**Keywords:** BNCT, PET, FBPA, FDG, Spatial correlation

## Abstract

**Background:**

^18^F-FDG PET is often utilized to determine BNCT selection due to the limited availability of ^18^F-BPA PET, which is performed by synthesizing ^18^F into the boron drug used for BNCT, although the uptake mechanisms between those are different. Additionally, only a few non-spatial point parameters, such as maximum SUV (SUV_max_), have reported a correlation between those in previous studies. This study aimed to investigate the spatial accumulation pattern between those PET images in tumors, which would be expected to either show higher uptake on ^18^F-BPA PET or be utilized in clinical, to verify whether ^18^F-FDG PET could be used as a selection indicator for BNCT.

**Methods:**

A total of 27 patients with 30 lesions (11 squamous cell carcinoma, 9 melanoma, and 10 rhabdomyosarcoma) who received ^18^F-FDG and ^18^F-BPA PET within 2 weeks were enrolled in this study. The ratio of metabolic tumor volumes (MTVs) to GTV, histogram indices (skewness/kurtosis), and the correlation of total lesion activity (TLA) and non-spatial point parameters (SUV_max_, SUV_peak_, SUV_min_, maximum tumor-to-normal tissue ratio (*T*_max_/*N*), and *T*_min_/*N*) were evaluated. After local rigid registration between those images, distances of locations at SUV_max_ and the center of mass with MTVs on each image and similarity indices were also assessed along its coordinate.

**Results:**

In addition to SUV_max_, SUV_peak_, and *T*_max_/*N*, significant correlations were found in TLA. The mean distance in SUV_max_ was $25.2 \pm 24.4\; {\text{mm}}$ and significantly longer than that in the center of mass with MTVs. The ratio of MTVs to GTV, skewness, and kurtosis were not significantly different. However, the similarities of MTVs were considerably low. The similarity indices of Dice similarity coefficient, Jaccard coefficient, and mean distance to agreement for MTV40 were $0.65 \pm 0.21$, $0.51 \pm 0.21$, and $0.27 \pm 0.30$ cm, respectively. Furthermore, it was worse in MTV50. In addition, spatial accumulation patterns varied in cancer types.

**Conclusions:**

Spatial accumulation patterns in tumors showed low similarity between ^18^F-FDG and ^18^F-BPA PET, although the various non-spatial point parameters were correlated. In addition, the spatial accumulation patterns were considerably different in cancer types. Therefore, the selection for BNCT using ^18^F-FDG PET should be compared carefully with using ^18^F-FBPA PET.

**Supplementary Information:**

The online version contains supplementary material available at 10.1186/s40658-022-00514-7.

## Background

Boron neutron capture therapy (BNCT) is an innovative radiation therapy that selectively destroys tumor cells using alpha and lithium particles generated from the ${}_{{}}^{10} {\text{B}}\left( {n,\alpha } \right){}_{{}}^{7} {\text{Li}}$ neutron capture reaction between thermal neutron and boron [1]. *Para*-boronophenylalanine (BPA) agent, specific for the L-type amino acid transporter 1 (LAT1) expressed in tumors, can selectively uptake the boron compounded into cancer, while the lower uptake into normal tissues is expected [2, 3]. In the current treatment planning of BNCT, the estimated dose distribution is generally derived from uniform tumor BPA uptake. The concentration is calculated based on a particular ratio to the blood concentration [4, 5]. Generally, the tissue-to-blood ratio of boron concentration in the tumor and brain was 3.5 and 1.0, respectively [5]. Therefore, heterogeneity uptake between tumors or cells is not considered in the current treatment planning of BNCT.

Fluoride-18-labeled (^18^F) BPA positron emission tomography (PET) enables visualization resembling BPA metabolism. The use of the distribution is one of the most optimal methods to determine the indication of BNCT [6–10]. A recent study suggested that the minimum count in tumor to the count in normal tissue ratio $\left( {T_{\min } /N} \right) \ge 2.5$ on ^18^F-BPA PET was a valuable selection indicator for recurrent head and neck squamous cell carcinoma, although the tumor-to-normal tissue ($T/N$) ratio, which reflected heterogeneity uptake insufficiently, had been considered [11]. Additionally, the previous study also suggested that the estimated dose distribution derived from uniform tumor BPA uptake did not correlate with the clinical outcome in BNCT [12]. Therefore, it is crucial for the selection indicators for BNCT that the spatial uptake information is considered on ^18^F-BPA PET. However, although BNCT for unresectable locally advanced or locally recurrent head and neck cancer has been covered by public health insurance in Japan since 2020, the requirements for insurance treatment do not include ^18^F-BPA PET [13].

Several studies reported the relationship of PET-based indices between ^18^F-fluorodeoxyglucose (FDG) and ^18^F-BPA PET to investigate a surrogate indicator because the ^18^F-BPA PET was available only in limited institutions. Igaki et al. suggested that the maximum standardized uptake value (SUV_max_) between ^18^F-BPA and ^18^F-FDG PET showed a high correlation among SUV_max_, TNR, and tissue-to-blood ratio [14]. Furthermore, Tani et al. performed receiver operating characteristics analysis and reported that ${\text{SUV}}_{{{\text{max}}}} \ge 5$ on ^18^F-FDG PET is suggestive of high ^18^F-BPA accumulation [15].

However, those indicators did not sufficiently reflect the spatial uptake information of the boron compound because SUV_max_ was non-spatial point information. In addition, ^18^F-FDG lacks specificity for malignant tumors because it shows false-positive accumulation such as inflammation and benign tumors [16, 17]. Differences between the spatial ^18^F-BPA and ^18^F-FDG uptake could affect the accuracy of the estimated radiation dose to the tumor and normal tissue. It might be inappropriate to use ^18^F-FDG PET as the selection indicator for BNCT.

Kobayashi et al. analyzed the voxel-by-voxel spatial correlation of SUVs within tumors of ^18^F-FDG and ^18^F-BPA PET using a deformable image registration technique in 11 head and neck cancer patients [18]. They then reported that the spatial distribution of SUVs within tumors was significantly positively correlated in 9/10 patients. However, their study focused on only head and neck cancer patients. Moreover, there were no similarity and heterogeneity evaluations in a metabolically active tumor volume, which was expected to be related to therapeutic response and prognosis prediction, although the spatial correlation of SUV in the entire tumor was evaluated. The purpose of this study was to compare the intratumoral spatial distribution between ^18^F-FDG and ^18^F-BPA PET using several non-spatial and spatial parameters for squamous cell carcinoma, melanoma, and rhabdomyosarcoma, which would be expected to either show higher uptake on ^18^F-BPA PET or utilize BNCT in the clinic, to verify the applicability whether ^18^F-FDG PET could be utilized for selection indicator for BNCT.

## Materials and methods

### General

This retrospective study, in which data had been derived from a previous prospective study [15], was approved by the institutional review board (approval number, 2017-091) of National cancer center hospital, Tokyo, Japan, and all patients signed informed consent.

### Patients

Patient characteristics are summarized in Table 1. A total of 27 patients diagnosed histologically with squamous cell carcinoma (SCC), melanoma (Mel), and rhabdomyosarcoma (RS) (17 males and 9 females, median age 45 years, age range 8–72) were enrolled in this study. PET examinations were performed from June 2012 to July 2016. Eleven (11 lesions) of 26 patients had SCC, 7 (9 lesions) had Mel, and 8 (10 lesions) had RS. The primary sites of SCC patients were tongue (4 patients), nasopharyngeal (1 patient), oropharyngeal (2 patients), hypopharyngeal (1 patient), external ear (2 patients), and nasal cavity (1 patient). In this study, the selected cancer types have been reported to have high expression of the LAT1 transporter (Mel/RS), which is involved in BPA uptake or to acquire the favorable clinical outcome through the clinical trials of BNCT (SCC), to be analyzed in the tumor which may be candidates tumor for BNCT [19, 20]. Thus, accumulation in tumors on ^18^F-BPA PET is expected. Then, a total of 30 lesions (SCC 11 lesions, Mel 9 lesions, and RS 10 lesions) were analyzed, excluding 2 lesions. One of them was a small cervical tumor (0.56 cc) surrounded by physiological muscle accumulation, which was expected to underestimate SUV_max_ due to partial volume effect. The other was the tumor nearby bladder, which excretes BPA in the urine, and had a higher urine radioactivity accumulation, affecting the analysis.

**Table 1:** Patient’s characteristics

Characteristics		
Age	Median (range)	45 (8–72) years
Sex	Male: Female	17: 9
Weight	Mean (range)	54.0 (19–95) kg
Administration	FDG: FBPA	206.1 MBq: 231.8 MBq

### PET/CT examination

Whole-body ^18^F-FDG and ^18^F-BPA PET/CT examinations were performed using Discovery 600 PET/CT scanner (GE Healthcare, Milwaukee, WI, USA). For both PET scans, the scan range was set from the top of the skull to the knee. PET detectors consisted of 12,288 *bismuth germanium oxide* crystal arrays with a dimension of 4.7 × 6.3 × 30 mm^3^. The axial field of view (FOV) was 153 mm, and the transaxial FOV was 700 mm. PET slice thickness was 3.27 mm, and consequently, 47 slices can be obtained with one bed position. The coincidence timing window was 9 ns. Detailed PET/CT image acquisition parameters and reconstruction methods were shown in the previous report on clinical trials [15].

**Fig. 1:**
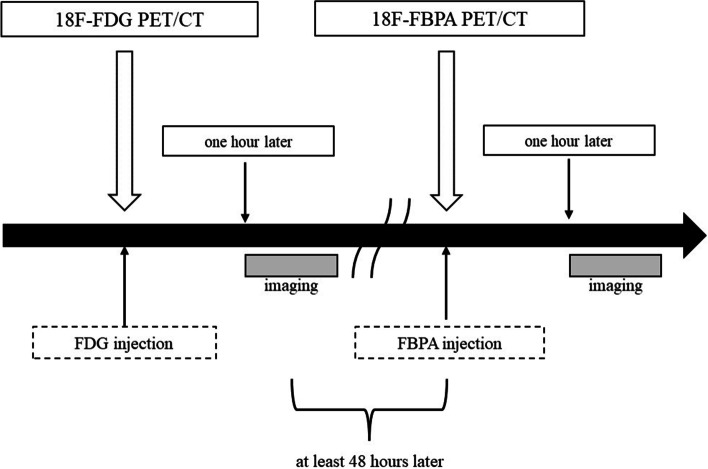
The schedule of ^18^F-FDG PET/CT and ^18^F-BPA PET/CT examination

Patients were examined for ^18^F-FDG PET/CT and ^18^F-BPA PET/CT within 2 weeks according to the schedule shown in Fig. 1. For ^18^F-FDG PET/CT examination, patients fasted for at least 4 h to promote uptake of ^18^F-FDG before the scheduled injection. The injected radioactivity of ^18^F-FDG and ^18^F-BPA was approximately 4.0 MBq/kg. Images acquisitions were performed 60 min after the intravenous bolus injection of each radiopharmaceutical agent.

### ***Image registration and analysis between ***^***18***^***F-BPA and ***^***18***^***F-FDG PET***

**Fig. 2:**
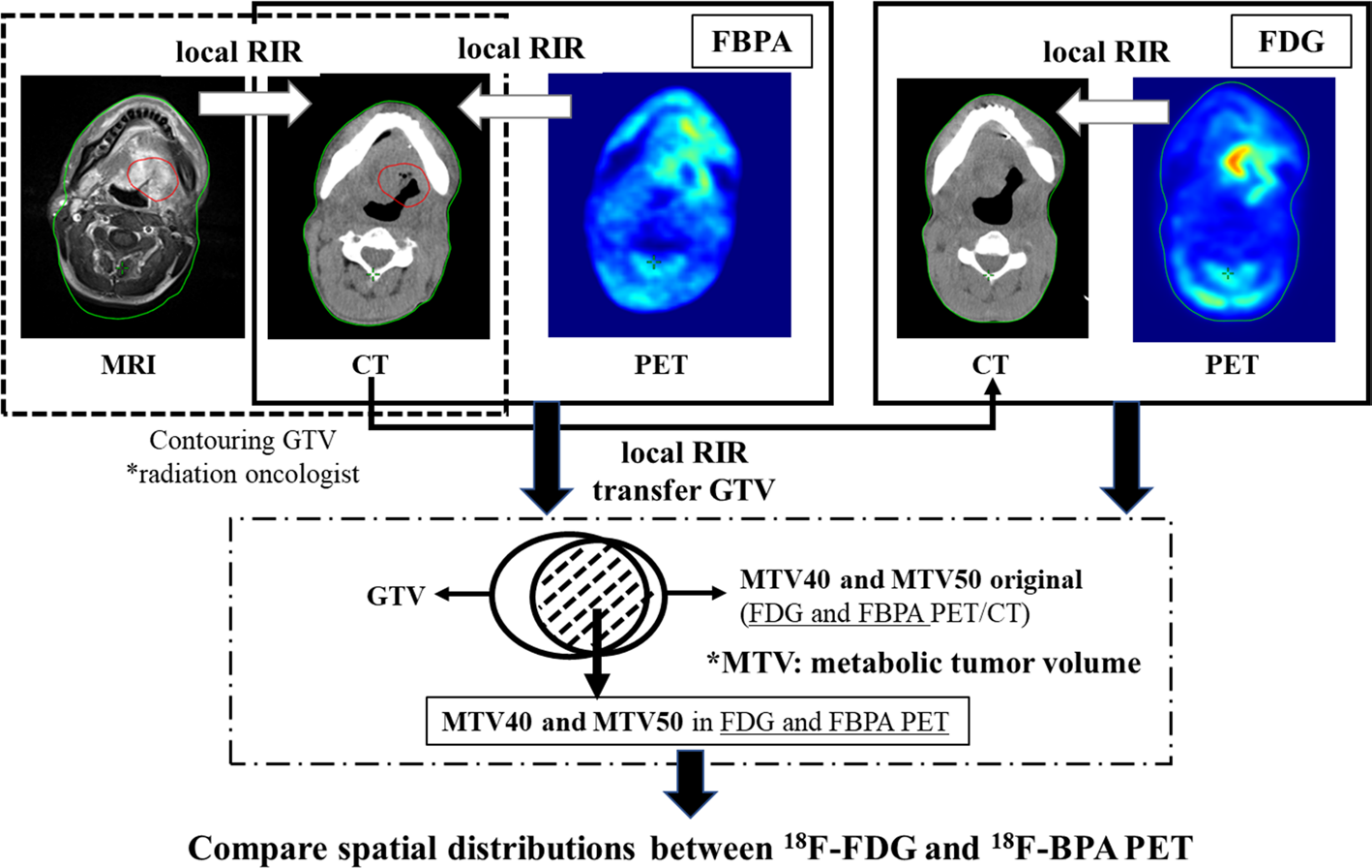
The process of image registration and analysis incorporated in the MIM workflow function

To compare the spatial accumulation between ^18^F-FDG and ^18^F-BPA PET, the image registration and analysis were performed using MIM maestro version 7.1.4 (MIM Software Inc., Cleveland, OH). The process of the image registration and the analysis incorporated in the MIM workflow function is shown in Fig. 2.

Initially, gross tumor volume (GTV) was delineated by one radiation oncologist on the CT images of the ^18^F-BPA PET/CT scan using a radiation treatment planning system (Eclipse version 15.6, Varian Medical Systems, Palo Alto, CA). In Table 2, GTVs and their equivalent diameters in all patients, SCC, Mel, and RS were shown. The equivalent diameter was defined as the diameter required for a sphere to have the same volume as the GTV. If possible, images from other modalities (e.g., contrast enhanced CT, magnetic resonance imaging) were used as references for delineating GTV after performing a local rigid image registration (RIR) on the CT image of ^18^F-BPA PET/CT. Then, local RIR was performed between PET and CT for ^18^F-FDG PET/CT and ^18^F-BPA PET/CT, respectively, focusing on the area around the GTV, and manual adjustments were referred to body contour and the contrast of surrounded normal tissue activities on PET image. Finally, local RIR was performed between CT images of ^18^F-FDG PET/CT and ^18^F-BPA PET/CT to match the structure around the GTV.

**Table 2:** GTVs and its diameters calculating from sphere formula in all patients, SCC, Mel, and RS

	GTV (cc)	Diameters of GTV (mm)
All patients (*n* = 30)	100.0 ± 136.5	57.6 ± 63.8
SCC (*n* = 11)	55.5 ± 51.5	47.4 ± 46.2
Mel (*n* = 9)	130.0 ± 219.5	62.8 ± 74.8
RS (*n* = 10)	121.9 ± 102.0	61.5 ± 57.9

*GTV* gross tumor volume, *SCC* squamous cell carcinoma, *Mel* melanoma, *RS* rhabdomyosarcoma

### ***Compare the spatial accumulation between ***^***18***^***F-FDG and ***^***18***^***F-BPA PET***

Metabolic tumor volumes (MTVs), which indicate the PET tracer accumulation region, were determined in each PET image. MTV is defined as the region on the PET image with an SUV greater than a threshold SUV, calculated by multiplying SUV_max_ by an arbitrary percentage. In this study, we used MTV calculated by 40% (MTV40) and 50% (MTV50). MTV40 and MTV50, excluding areas outside the GTV, were used for the final comparison between ^18^F-FDG PET and ^18^F-BPA PET images. The cutoff value for MTV is calculated from the SUV_max_, which is the reported correlation between the two tracers. It would be meaningful to compare the spatial correspondence and heterogeneity of MTVs in relation to the therapeutic response and prognostic prediction between ^18^F-FDG and ^18^F-BPA PET because MTVs in ^18^F-BPA expect the relative high accumulation of BPA and favorable therapeutic response in BNCT. To quantitatively evaluate differences in spatial accumulation patterns, we conducted the following six evaluations between ^18^F-FDG and ^18^F-BPA PET.

1. The correlation of SUV_max_, minimum SUV (SUV_min_), peak SUV (SUV_peak_), and T/N ratios: The correlation of non-spatial point parameters between ^18^F-FDG and ^18^F-BPA PET was assessed to compare representative accumulation points. SUV_peak_ was determined as the highest mean SUV measured using a 1 cm^3^ sphere volume of interest including SUV_max_. This value can reduce image noise’s effect, mainly due to the imaging and reconstruction parameters. To consider the heterogeneity in tumors, two tumor-to-normal ratios (*T*_max_/*N* and *T*_min_/*N*) were calculated from SUV_max_ and SUV_min_ within the tumor, respectively. The value of normal tissue was determined by the average of three circular region-of-interest (diameter; 1 cm) around the GTV.
2. The correlation of total lesion activity (TLA) within GTV, MTV40, and MTV50: In addition to the point correlations mentioned above, the correlation of TLA between ^18^F-FDG and ^18^F-BPA PET was investigated. The TLA, which was the non-spatial volumetric parameter, was defined as the multiplication of the specific volume (GTV, MTV40, and MTV50) by each SUV_mean_. TLA in ^18^F-BPA PET can indicate a similar value for the amount of BPA despite differences in injection methods and amounts of pharmaceutics. The volumetric evaluation can be performed without the influence of image noise.
3. The distances (mm) between locations at SUV_max_ and the center of mass with MTV40 and MTV50 in ^18^F-FDG and ^18^F-BPA PET: It was automatically calculated by matching the coordinates of registered each PET image by MIM maestro’s workflow function. The distance between locations at SUV_max_ was chosen to verify whether the SUV_max_ correlations reported in previous studies assessed the same spatial accumulation points between ^18^F-FDG and ^18^F-BPA PET. In addition, the distances between the center of mass with MTVs were evaluated to verify the spatial location of MTVs between the two PET tracers.
4. The volume ratios of MTV40 and MTV50 to GTV: The non-spatial parameters representing the ratio of accumulation (MTV40 and MTV50) to whole GTV were compared between ^18^F-FDG and ^18^F-BPA PET. These non-spatial parameters were chosen to clarify the volume ratio of high-accumulation areas based on SUV_max_ for GTV between ^18^F-FDG and ^18^F-BPA PET.
5. The similarity indices of MTV40 and MTV50: Three similarity indices between ^18^F-FDG and ^18^F-BPA PET, including Dice similarity coefficient (DSC), Jaccard coefficient (JC), and mean distance to agreement (MDA, cm), were calculated for MTV40 and MTV50. The equation of Boolean operation of DSC and JC was as follows
  $${\text{DSC}}\left( {{\text{MTV}}_{{{\text{FDG}}}} ,{\text{MTV}}_{{{\text{FBPA}}}} } \right) = \frac{{2\left| {{\text{MTV}}_{{{\text{FDG}}}} \cap {\text{MTV}}_{{{\text{FBPA}}}} } \right|}}{{\left| {{\text{MTV}}_{{{\text{FDG}}}} } \right| + \left| {{\text{MTV}}_{{{\text{FBPA}}}} } \right|}}$$

and

$${\text{JC}}\left( {{\text{MTV}}_{{{\text{FDG}}}} ,{\text{MTV}}_{{{\text{FBPA}}}} } \right) = \frac{{\left| {{\text{MTV}}_{{{\text{FDG}}}} \cap {\text{MTV}}_{{{\text{FBPA}}}} } \right|}}{{\left| {{\text{MTV}}_{{{\text{FDG}}}} \cup {\text{MTV}}_{{{\text{FBPA}}}} } \right|}},$$

respectively, where MTV_FDG_ and MTV_FBPA_ are MTVs obtained from ^18^F-FDG and ^18^F-BPA PET, respectively. MDA represents the mean distance within a shortest distance that points on the contour of Boolean MTV_FDG_ (Boolean MTV_FBPA_) can reach any point on the contour of Boolean MTV_FBPA_ (Boolean MTV_FDG_). MDA can be calculated using the following equation:

$${\text{MDA}}\left( {A, B} \right) = {\text{mean}}_{a} \in_{A,b} \in_{B} \left\{ {d\left( {a, B} \right) \cup d\left( {b, A} \right)} \right\},$$

where *a* and *b* represent any point at the outlines of structures *A* (MTV on ^18^F-FDG) and *B* (MTV on ^18^F-BPA) and *d* (*a*, *B*) denotes the minimal distance between point *a* and any point in structure *B* and vice versa. If the outlines of the two structures are completely consistent, the MDA is zero. Then, DSC and JC between MTV40 and MTV50 were compared to evaluate the accumulation heterogeneity in ^18^F-FDG and ^18^F-BPA PET. In addition, MDA was used to quantify differences in the spatial location of MTV40 and MTV50 between the two tracers. These parameters were chosen to investigate the spatial correlation of MTVs between ^18^F-FDG and ^18^F-BPA PET.

6. The histogram indices of GTV, MTV40, and MTV50: Two histogram indices, including skewness and kurtosis, were calculated from SUV distribution within GTV, MTV40, and MTV50 and were compared between ^18^F-FDG and ^18^F-BPA PET. These parameters were chosen to evaluate differences in the heterogeneity of accumulation in MTVs rather than the spatial correlation of it. Skewness and kurtosis were the first-order radiomics features which were related to tumor response and prognosis prediction.

### Statistics

The correlations between SUV_max_, SUV_peak_, SUV_min_, *T*_max_/*N*, *T*_min_/*N*, and TLA ^18^F-FDG PET and those in ^18^F-BPA PET were evaluated using the Pearson correlation coefficient. We defined the strength of the correlation according to *r* as follows: $r \ge 0.9$ as very strong, $0.9 > r \ge 0.7$ as strong, $0.7 > r \ge 0.5$ as mild, $0.5 > r \ge 0.3$ as weak, and $0.3 > r$ as none. In addition, the test of no correlation was performed to exclude the indicators with no correlation. Wilcoxon singed ranked tests were performed for all combinations between each distance (SUV_max_ and the center of mass with MTV40 and MTV50), obtained from ^18^F-FDG and ^18^F-BPA PET. The same analysis was also performed between paired samples (the volume ratio and the histogram indices between ^18^F-FDG and ^18^F-BPA PET and the similarity indices between MTV40 and MTV50). For comparison of the distances between locations at SUV_max_, and the center of mass with MTV40 and MTV50 for the two PET tracers in each cancer type (SCC, Mel, RS), the Mann–Whitney U test was used. A *p* value of less than 0.05 was considered statistically significant and ranging from 0.05 to 0.10 was considered a statistical trend. Data were expressed as mean $\pm$ standard deviation. The distributions of the data in the figures were expressed by box plots. A box covers the 1st quartile, median, and 3rd quartile. A cross in the box is the mean value, and whiskers indicate the maximum and minimum value. The DSC and JC mean the overlapping volume ratio between the MTVs obtained from ^18^F-FDG and the MTVs obtained from ^18^F-BPA PET (ground truth). Their range is theoretically limited to a range of 0 to 1. All statistical analyses were performed using EZR (Saitama Medical Center, Jichi Medical University, Saitama, Japan) [21].

## Results

**Fig. 3:**
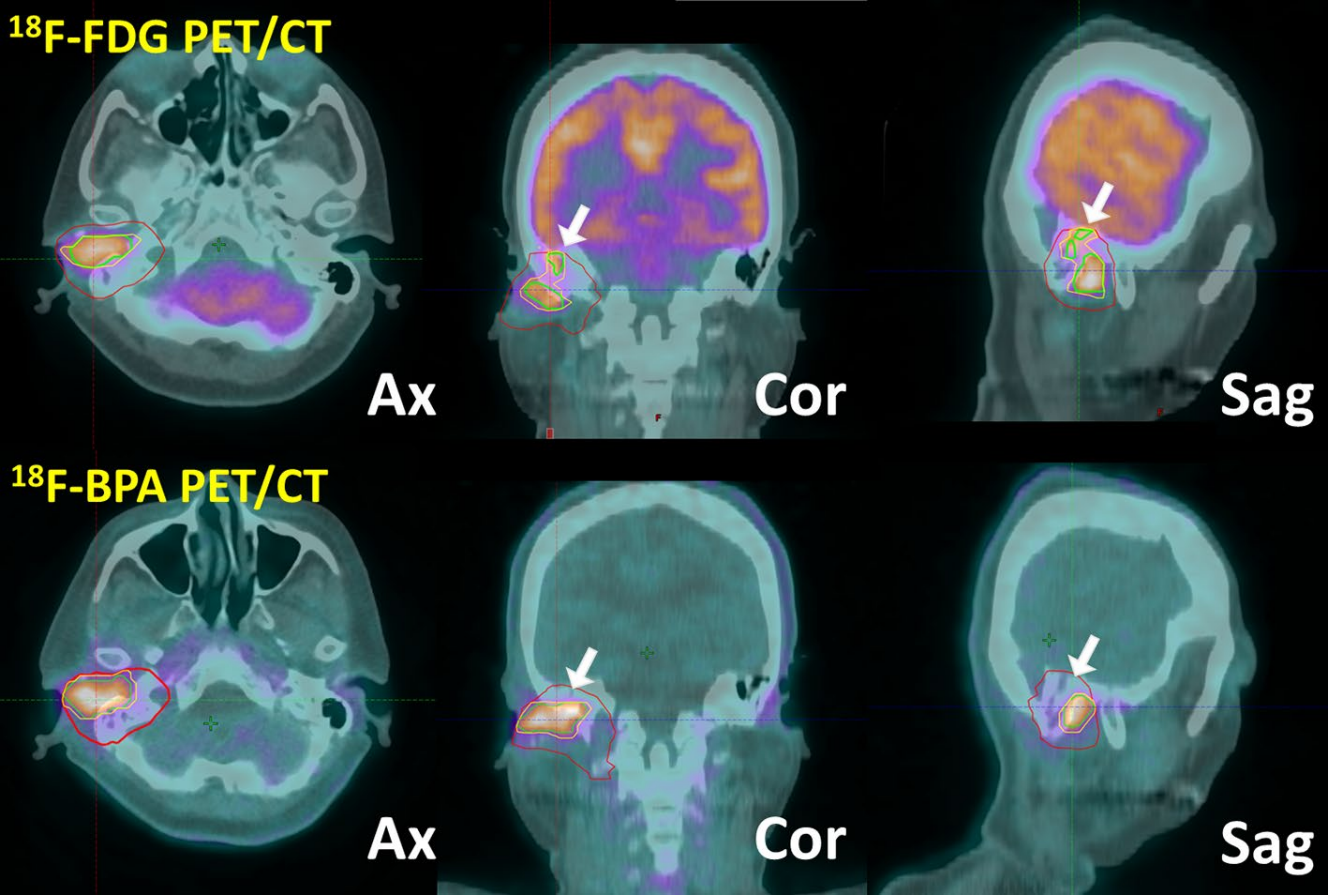
A 50-year-old woman was diagnosed with squamous cell carcinoma in the right external ear. The top and bottom images show fused images of ^18^F-FDG PET/CT and those of ^18^F-BPA PET/CT, respectively. The solid line with red, yellow, and green indicates the contour of GTV, MTV40, and MTV50, respectively. The discrepancy (white arrows) between two tracers is observed in coronal and sagittal planes due to physiological brain accumulation or inflammation (mastoiditis or external otitis)

Figure 3 indicates ^18^F-FDG and ^18^F-BPA fused images of a case with characteristic discrepancies in each MTVs. DSC, JC, and MDA with MTV40 were 0.60, 0.43, and 0.25 cm, respectively. Those with MTV50 were 0.56, 0.39, and 0.26 cm, respectively.

**Fig. 4:**
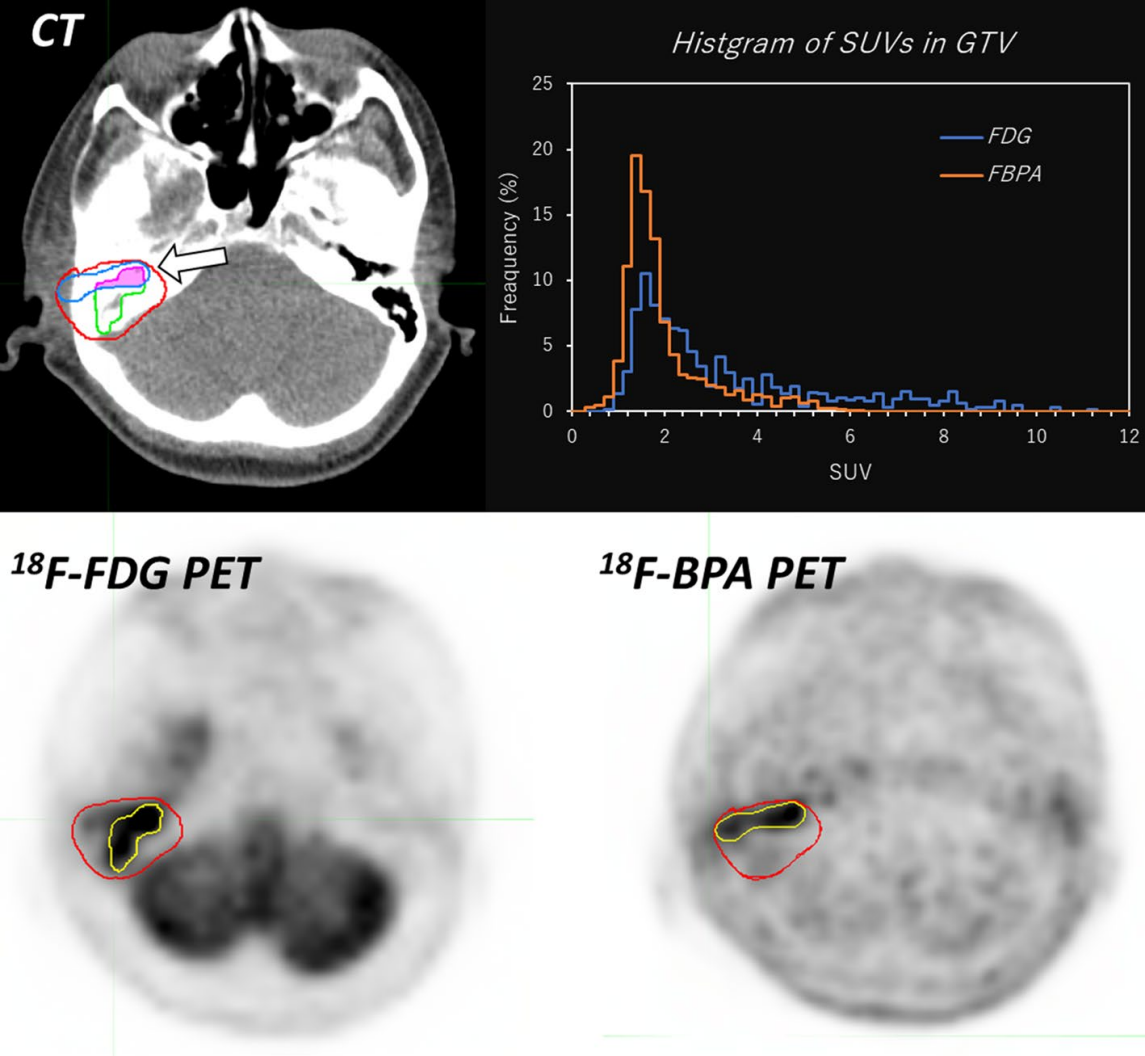
The example images of measure similarities (DSC, JC, and MDA) and histogram indices (skewness and kurtosis). The red and yellow lines on PET images (bottom images) show the contour of GTV and MTV40 on each PET image, respectively. The white arrow on the CT image indicates the overlap region between MTV40 on ^18^F-FDG and that on ^18^F-BPA PET. The overlap volume is used to calculate DSC and JC. The top right image shows histograms of SUVs derived from GTVs on both PET images for the calculation of skewness and kurtosis

Figure 4 shows the example images of measure similarities (DSC, JC, and MDA) and histogram indices (skewness and kurtosis).

### The correlation of non-spatial point parameters

**Fig. 5:**
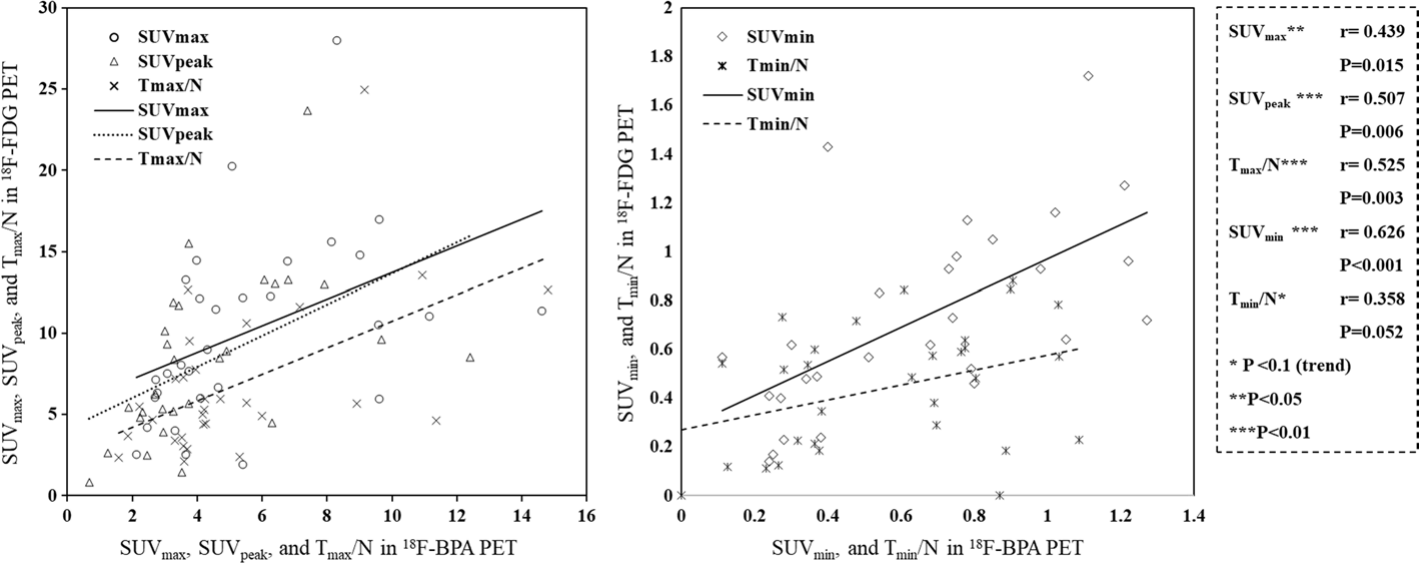
The correlation of non-spatial point parameters, including SUV_max_, SUV_peak_, SUV_min_, *T*_max_/*N*, and *T*_min_/*N* between ^18^F-FDG and ^18^F-BPA PET for all patients. *T*_max_/*N*; maximum tumor-to-normal tissue count ratio, *T*_min_/*N* minimum tumor-to-normal tissue count ratio

Figure 5 shows the correlations of SUV_max_, SUV_peak_, SUV_min_, *T*_max_/*N*, and *T*_min_/*N* between ^18^F-FDG and ^18^F-BPA PET for all patients. SUV_peak_, SUV_min_, and *T*_max_/*N* showed a mild correlation between ^18^F-FDG and ^18^F-BPA PET (*r* = 0.507; *P* = 0.006, *r* = 0.626; *P* < 0.001, and *r* = 0.525; *P* = 0.003, respectively). On the other hand, SUV_max_ and *T*_min_/*N* showed a weak correlation (*r* = 0.439; *P* = 0.015 and *r* = 0.358; *P* = 0.052, respectively). However, the correlation of non-spatial point parameters varied by the cancer types. For SCC, SUV_max_ and *T*_max_/*N* showed a strong correlation between ^18^F-FDG and ^18^F-BPA PET (*r* = 0.726; *P* = 0.011 and *r* = 0.718; *P* = 0.013, respectively, Additional file 1: Fig. S1A). On the other hand, Mel showed a strong correlation in SUV_min_ (*r* = 0.703; *P* = 0.052, Additional file 2: Fig. S1B). RS showed a strong correlation in SUV_max_, SUV_peak_, and SUV_min_ (*r* = 0.842; *P* < 0.002, *r* = 0.969; *P* < 0.001, and *r* = 0.862; *P* = 0.003, respectively, Additional file 3: Fig. S1C).

### The correlation of TLA within GTV, MTV40, and MTV50

**Fig. 6:**
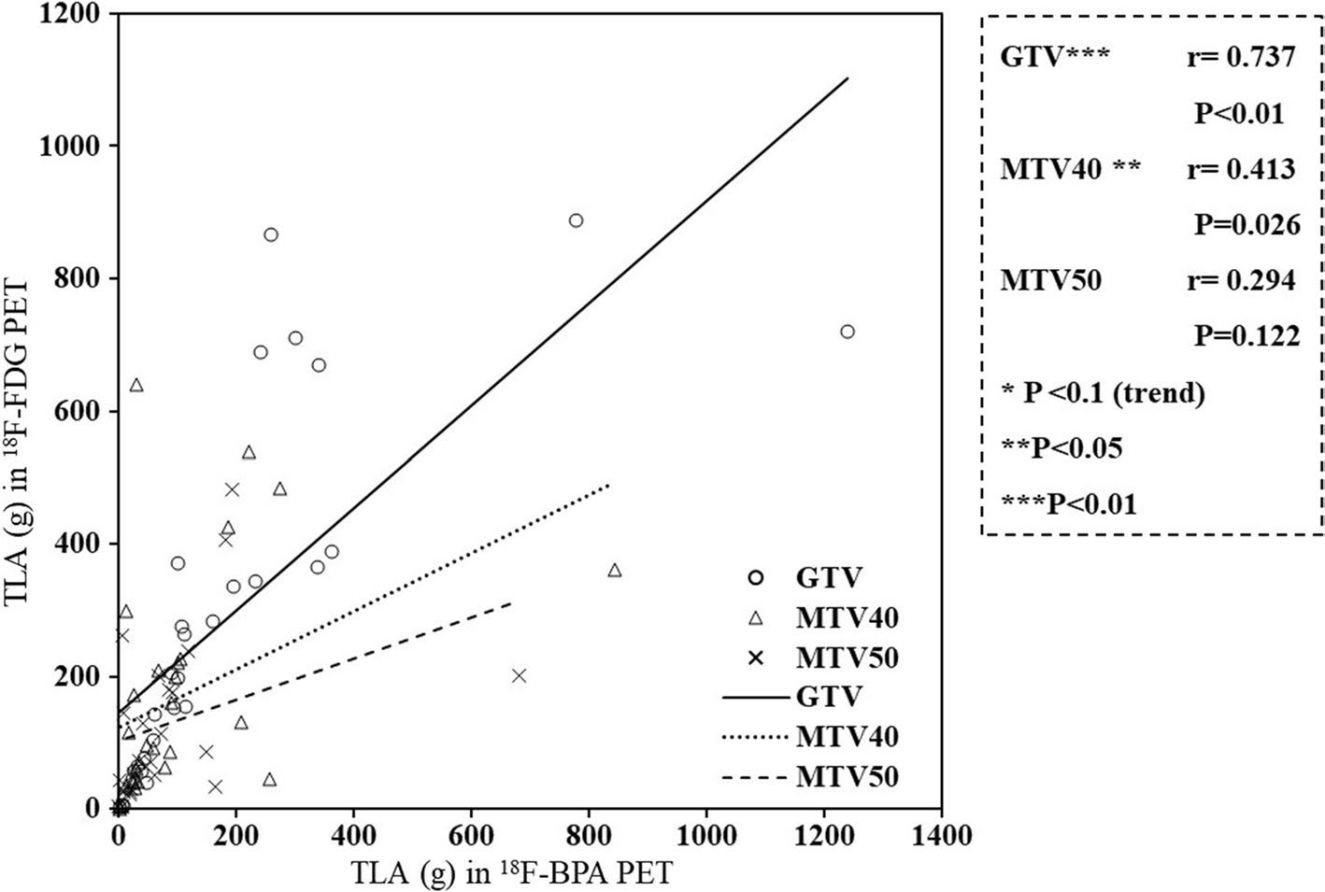
The correlation of TLA in GTV, MTV40, and MTV50 between ^18^F-FDG and ^18^F-BPA PET for all patients. TLA; total lesion activity

Figure 6 shows the correlations of TLAs within GTV, MTV40, and MTV50 between ^18^F-FDG and ^18^F-BPA PET for all patients. The TLAs within GTV showed a strong correlation between ^18^F-FDG and ^18^F-BPA PET (*r* = 0.737; *P* < 0.01), while those within MTV40 showed the weak correlation (*r* = 0.413; *P* = 0.026). The correlation of TLAs varied by the cancer types (Additional files 4, 6: Fig. 2A, C). For SCC, TLAs within GTV, MTV40, and MTV50 showed a strong to very strong correlations between ^18^F-FDG and ^18^F-BPA PET (*r* = 0.921; *P* < 0.01, *r* = 0.831; *P* < 0.01, and *r* = 0.847; *P* < 0.01, respectively). For Mel, TLAs within GTV showed a strong correlations between ^18^F-FDG and ^18^F-BPA PET (*r* = 0.884; *P* < 0.01). For RS, TLAs within GTV showed a strong correlations (*r* = 0.808; *P* < 0.01), while TLAs within MTV50 showed a mild correlation (*r* = 0.580; *P* = 0.08).

### ***The distance between locations at SUV***_***max***_*** and the center of mass with MTV40 and MTV50 in ***^***18***^***F-FDG and ***^***18***^***F-BPA PET***

**Fig. 7:**
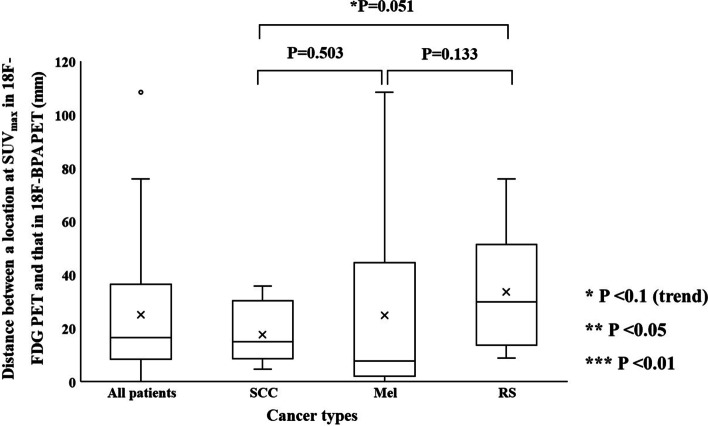
The distances between locations at SUV_max_ between ^18^F-FDG and ^18^F-BPA PET for all patients, squamous cell carcinoma, melanoma, and rhabdomyosarcoma

**Fig. 8:**
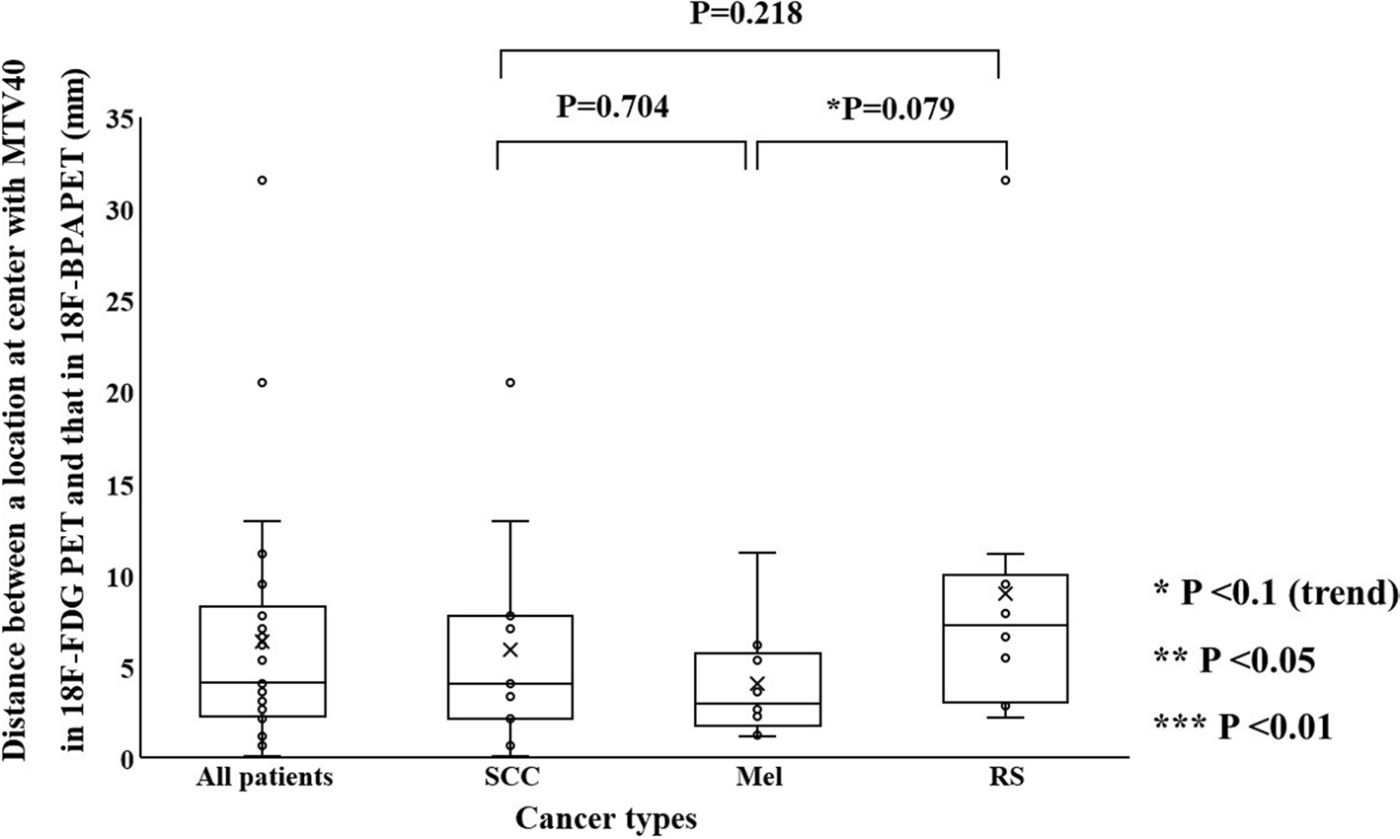
The distances between locations at the center of mass with MTV40 between ^18^F-FDG and ^18^F-BPA PET for all patients, squamous cell carcinoma, melanoma, and rhabdomyosarcoma

**Fig. 9:**
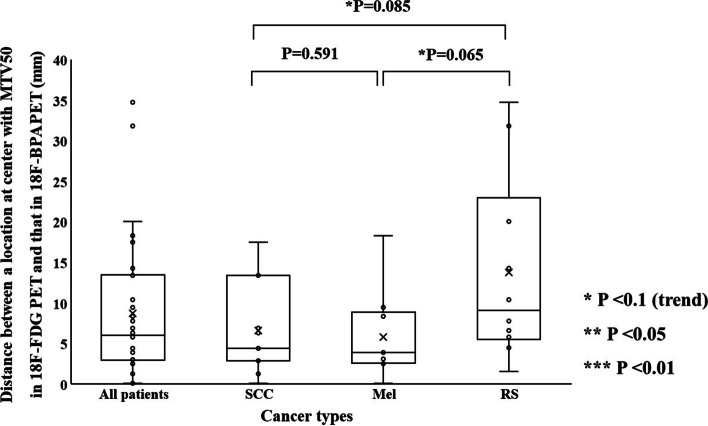
The distances between locations at the center of mass with MTV50 between ^18^F-FDG and ^18^F-BPA PET for all patients, squamous cell carcinoma, melanoma, and rhabdomyosarcoma

Figure 7 shows the distances at SUV_max_ between ^18^F-FDG and ^18^F-BPA PET in each cancer type. The mean distance between locations at SUV_max_ for SCC, Mel, and RS was $17.7 \pm 11.1\;{\text{mm}}$, $24.9 \pm 36.0\;{\text{mm}}$, and $33.6 \pm 21.9\;{\text{mm}}$, respectively. There were no statistically significant differences among cancer types. However, SCC tends to be a lower value than RS. That at the center of mass with MTV40 for SCC, Mel, and RS was $5.9 \pm 6.1$, $4.1 \pm 3.2$, and $9.0 \pm 8.5$ mm, respectively (Fig. 8). Mel tends to be a lower value than RS. That at the center of mass with MTV50 for SCC, Mel, and RS was $6.6 \pm 5.7$, $5.8 \pm 5.5$, and $13.8 \pm 11.6$ mm, respectively (Fig. 9). RS tends to be a higher in value than SCC and RS.

**Fig. 10:**
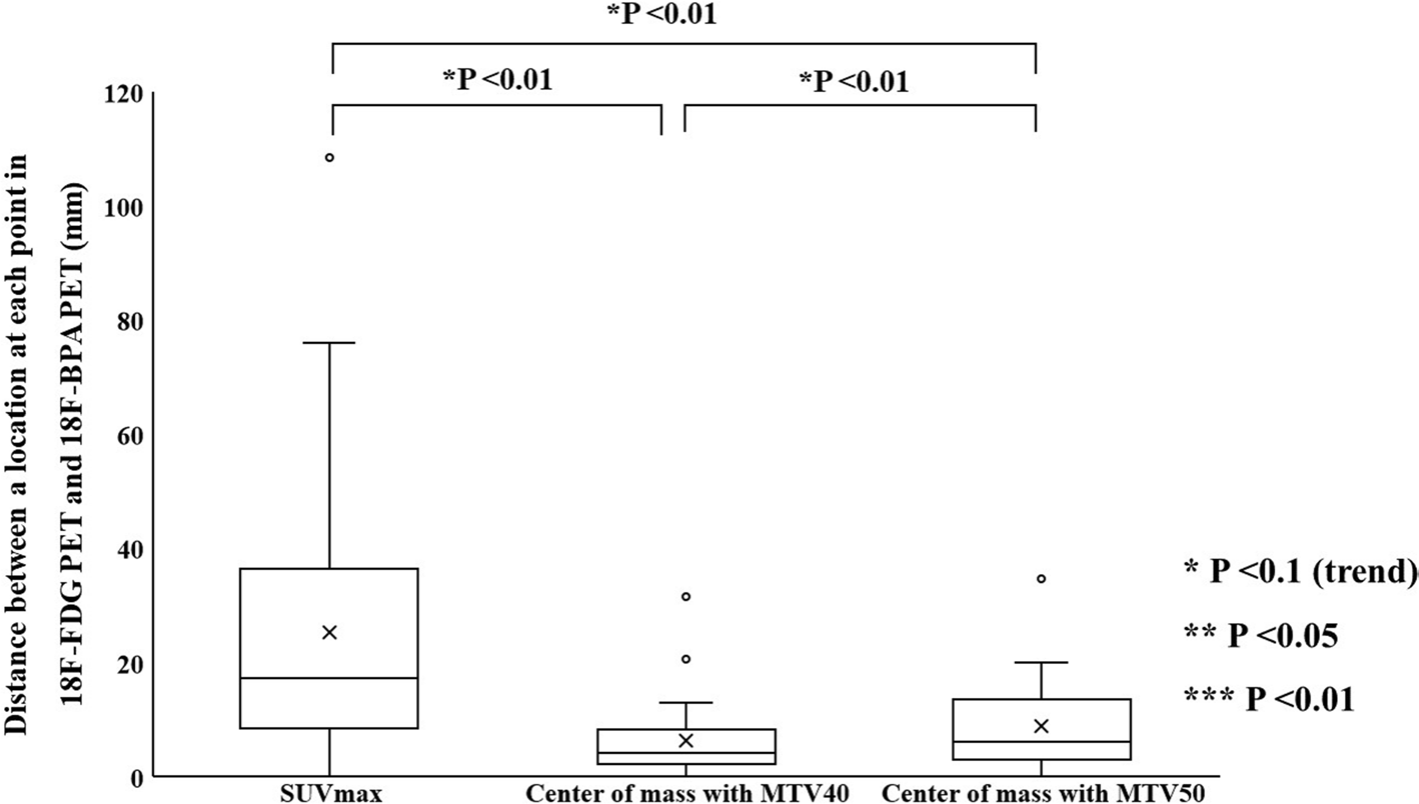
Comparison of all combinations between each distance (a location at SUV_max_, the center of mass with MTV40, and MTV50), obtained from ^18^F-FDG and ^18^F-BPA PET for all patients

Figure 10 shows the distances between locations at SUV_max_ and the center of mass with MTVs in ^18^F-FDG and ^18^F-BPA PET for all patients. The mean distance in SUV_max_, the center of mass with MTV40, and that MTV50 were $25.2 \pm 24.4\;{\text{mm}}$, $6.4 \pm 6.5$ mm, and $8.8 \pm 8.6$ mm, respectively. The distance in SUV_max_ was statistically significantly longer than that in the center of mass with each MTV. The distance in the center of mass with MTV40 was statistically significantly shorter than that in the center of mass with MTV50.

### The volume ratios of MTV40 and MTV50 to GTV

**Fig. 11:**
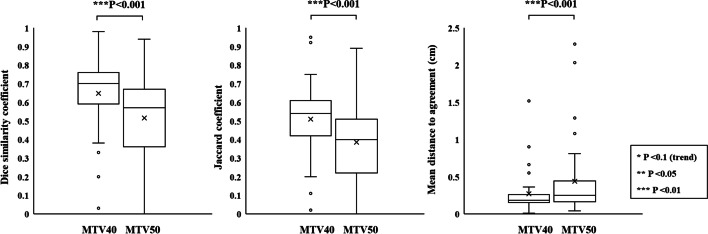
The volume ratio of MTV40 and MTV50 to GTV between ^18^F-FDG and ^18^F-BPA PET for all patients

Figure 11 shows the volume ratios of MTV40 and MTV50 to GTV in ^18^F-FDG and ^18^F-BPA PET for all patients. The volume ratios of MTV40 to GTV in ^18^F-FDG and ^18^F-BPA PET were $0.51 \pm 0.24$ and $0.55 \pm 0.27$, respectively. The volume ratios of MTV50 to GTV in ^18^F-FDG and ^18^F-BPA PET were $0.36 \pm 0.21$ and $0.39 \pm 0.26$, respectively. There were no statistically significant differences in the volume ratio of MTV40 to GTV and that of MTV50 to GTV between ^18^F-FDG and ^18^F-BPA PET. For SCC, the volume ratio of those MTVs to GTV in ^18^F-BPA PET shows statistically significant higher value than those in ^18^F-FDG PET (MTV40; *P* = 0.004, MTV50; *P* = 0.004, Additional file 7: Fig. S3).

### The similarity indices of MTV40 and MTV50

**Fig. 12:**
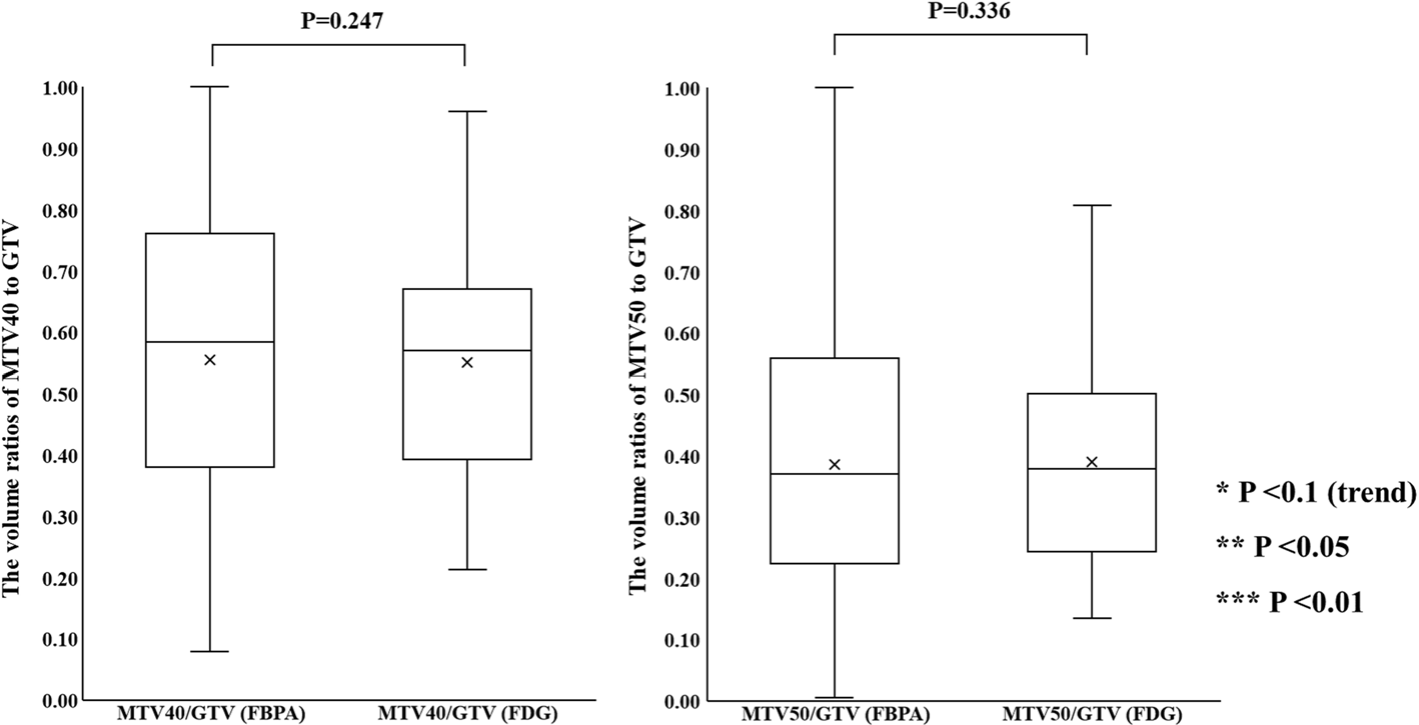
The similarity indices, including Dice similarity coefficient, Jaccard coefficient, and mean distance to agreement, of MTV40 and MTV50 between ^18^F-FDG and ^18^F-BPA PET for all patients

Figure 12 shows DSC, JC, and MDA of MTV40 and MTV50 between ^18^F-FDG and ^18^F-BPA PET for all patients. DSC between ^18^F-FDG and ^18^F-BPA PET was $0.65 \pm 0.21$ for MTV40 and $0.52 \pm 0.25$ for MTV50. JC between ^18^F-FDG and ^18^F-BPA PET was $0.51 \pm 0.21$ for MTV40 and $0.38 \pm 0.22$ for MTV50. MDA between ^18^F-FDG and ^18^F-BPA PET was $0.27 \pm 0.30\;{\text{cm}}$ for MTV40 and $0.44 \pm 0.54\;{\text{cm}}$ for MTV50. The DSC, JC, and MDA similarity indices of MTV40 and MTV50 were low. Furthermore, those in MTV50 show significantly worse values than those in MTV40. A similar tendency was found in each cancer type (Additional files 8, 9, 10: Fig. S4A–C).

### The histogram indices of GTV, MTV40, and MTV50

**Fig. 13:**
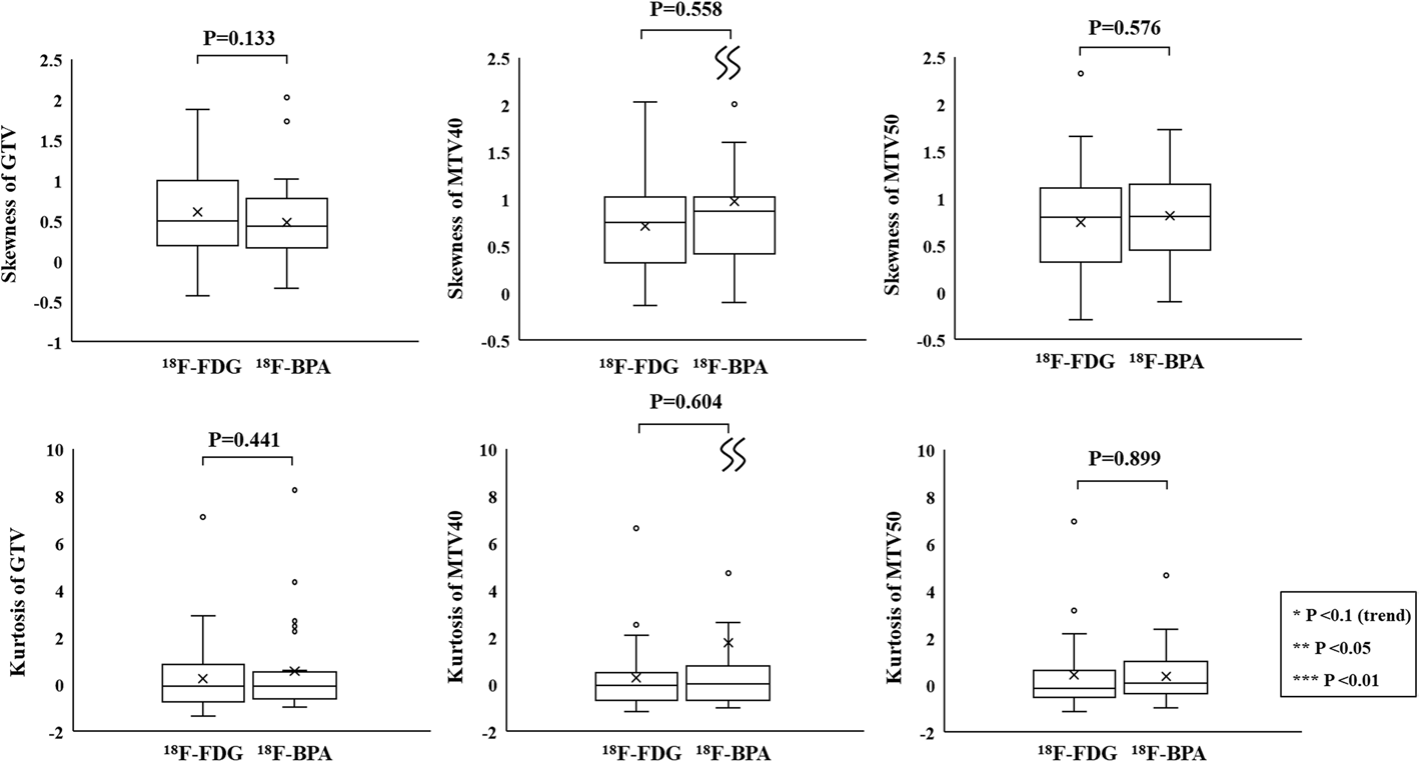
The histogram indices of all patients, including skewness and kurtosis, of GTV, MTV40, and MTV50 in ^18^F-FDG and ^18^F-BPA PET

Figure 13 shows the skewness and kurtosis of MTV40 and MTV50 in ^18^F-FDG and ^18^F-BPA PET for all patients. The skewness of GTV in ^18^F-FDG and ^18^F-BPA PET was $0.61 \pm 0.56$ and $0.48 \pm 0.55$, respectively. That of MTV40 in ^18^F-FDG and ^18^F-BPA PET was $0.71 \pm 0.51$ and $0.97 \pm 1.02$, respectively. That of MTV50 in ^18^F-FDG and ^18^F-BPA PET was $0.75 \pm 0.56$ and $0.82 \pm 0.44$, respectively. The kurtosis of GTV in ^18^F-FDG and ^18^F-BPA PET was $0.24 \pm 1.65$ and $0.55 \pm 1.95$, respectively. That of MTV40 in ^18^F-FDG and ^18^F-BPA PET was $0.26 \pm 1.53$ and $1.78 \pm 6.26$, respectively. That of MTV50 in ^18^F-FDG and ^18^F-BPA PET was $0.43 \pm 1.67$ and $0.38 \pm 1.21$, respectively. MTV40 and MTV50 showed no statistically significant differences in skewness and kurtosis between ^18^F-FDG and ^18^F-BPA PET. In the evaluation of each cancer type, SCC shows statistically significant differences in the skewness of GTV between ^18^F-FDG and ^18^F-BPA PET (*P* = 0.024, Additional file 11: Fig. S5A). RS shows statistically significant differences in skewness and kurtosis of MTV40 between ^18^F-FDG and ^18^F-BPA PET (*P* = 0.030 and 0.030, respectively, Additional file 13: Fig. S5C).

## Discussion

This study was the first report to perform a comprehensive comparison of the intratumoral spatial distribution between ^18^F-FDG and ^18^F-BPA PET using several non-spatial and spatial parameters for squamous cell carcinoma, melanoma, and rhabdomyosarcoma, which would be expected to either show higher uptake on ^18^F-BPA PET or utilize in the clinic, to verify whether ^18^F-FDG PET could be utilized for selection indicator for BNCT. Due to the limitation of the availability of ^18^F-BPA, several studies have already been reported to use the non-spatial point parameter, such as SUV_max_, derived from ^18^F-FDG as a surrogate selection indicator instead of ^18^F-BPA PET [14, 15]. However, the previous studies also suggested that the evaluation using SUV_max_ alone would not reflect the spatial location and the heterogeneity of BPA uptake sufficiently [12] since the PET tracer in ^18^F-FDG and ^18^F-BPA has different metabolic patterns [14, 22, 23]. This study evaluated not only the correlation of various non-spatial point parameters (SUV_max_, SUV_peak_, SUV_min_, *T*_max_/*N*, and *T*_min_/*N*), but also TLA, the distances of SUV_max_ and the center of mass with MTVs, the volume ratios of MTVs to GTV, and the similarity indices of MTVs between ^18^F-FDG and ^18^F-BPA PET in SCC, Mel, and RS. Additionally, we compared the heterogeneity of the SUV within the GTV and MTV. As a result, in addition to the correlation of non-spatial point parameters other than SUV_max_, this study indicated a discrepancy in the spatial location in the high-accumulation area (MTVs). Moreover, because the spatial accumulation pattern depends on the cancer types, it should be more careful in the case of using ^18^F-FDG PET as a surrogate indicator for BNCT.

The correlation of non-spatial point parameters for all patients showed statistically significant weak-to-mild correlations for SUV_max_, SUV_peak_, SUV_min_, and *T*_max_/*N* between ^18^F-FDG and ^18^F-BPA PET. However, our results for the comparisons among the cancer types indicated that SCC and RS had a significant correlation for high-accumulation points (SUV_max_ and SUV_peak_), while RS was also significantly correlated in SUV_min_. The previous studies reported only the correlation of SUV_max_ in non-spatial point parameters [14, 15]. Tani et al. reported the correlation coefficient of 0.72 for SUV_max_ between ^18^F-FDG and ^18^F-BPA PET in analyzing 20 head and neck cancer patients, including various cancer types [15]. Igaki et al. conducted a similar research for 82 patients in five cancer types, including SCC and Mel, and found a correlation coefficient of 0.4825 for all patients (SCC; *r* = 0.5957, Mel; *r* = 0.5632, range; − 0.1288–0.5957) [14]. Although slightly different correlation coefficients for all patients have been reported in previous studies, our results were consistent with those studies. On the other hand, in the analysis of different cancer types, different correlation coefficients were obtained in SCC and Mel patients. These results would be affected by differences in sample size. However, they may not rule out the divergence in the correlation coefficients among cancer types in our study. The purpose of investigating the correlation of each parameter is to examine whether an alternative value of ^18^F-FDG that corresponds to the value of ^18^F-BPA associated with the clinical outcome can be adequately utilized for the clinical indicator in BNCT. Therefore, using SUV_min_, which has a small range of value relative to the data reproducibility, may be inadequate, although significant correlations were observed in all patients and RS. Another study investigated the effect of inhomogeneous distribution in ^18^F-BPA PET for predicting the treatment effect of BNCT for recurrent head and neck squamous cell carcinoma, *T*_min_/*N* in ^18^F-BPA PET distinguished complete response (CR) and non-CR groups [11]. However, our study showed no correlation of *T*_min_/*N* between ^18^F-FDG and ^18^F-BPA PET. This result may imply the difficulty of predicting the treatment effects of head and neck SCC patients using *T*_min_/*N* in ^18^F-FDG PET.

Interestingly, TLAs within GTV between ^18^F-FDG and ^18^F-BPA showed a strong correlation regardless of cancer type, while the correlation in TLAs within MTVs was lower than that within GTV. The discrepancy between the correlation of the accumulation in the entire tumor and that in MTVs may imply heterogeneity of the accumulation within the tumor. Compared to SUV_max_, TLA has the advantages of enabling the evaluation of the amount of drug in the tumor and being less affected by image noise due to image reconstruction and imaging conditions. However, TLA is strongly influenced by volume due to the nature of its calculation method. Therefore, the results of correlation analysis in Mel may be unreliable due to the high variability of TLA. In addition to cancer type, studies that consider tumor stage and progression may provide a more helpful alternative to non-spatial point parameters.

The spatial relationship at the location of SUV_max_ and the center of mass with MTVs between ^18^F-FDG and ^18^F-BPA PET has not been sufficiently evaluated in previous reports. These indices were assessed to support the possibility that the SUV_max_ correlations evaluated in previous studies may be assessing different accumulation points within the tumor. Our study showed the distance between locations at SUV_max_ in ^18^F-FDG and ^18^F-BPA PET was $25.2 \pm 24.4\;{\text{mm}}$ for all patients, and this value was significantly larger than the distance in the center of mass with each MTV. Also, each evaluation of cancer types did not show significant differences in the spatial distance between them, although some statistical trends were observed in cancer types. These results suggested that the locations of SUV_max_ in ^18^F-FDG and ^18^F-BPA PET had a spatial difference larger than those of a center of the mass in the MTVs. The previous study investigating deformable registration accuracy between those PET images indicated high geometric accuracy, with surface distance and surface coverage errors of less than 1.5 mm [18]. Therefore, our result indicates that the correlation of SUV_max_ between ^18^F-FDG and ^18^F-BPA PET only evaluates the maximum activity at different spatial points and, more specifically, in other cells. Furthermore, because the evaluation between single voxels would be susceptible to image noise [24, 25] and image registration accuracy [26], we evaluated the spatial relationship between MTVs in ^18^F-FDG and ^18^F-BPA PET. As a result, there were no statistically significant differences in the volume ratio of MTVs to GTV between ^18^F-BPA and ^18^F-FDG PET. However, the similarity indices of MTV40 and MTV50 were low. Those values including DSC, JC, and MDA in MTV40 between ^18^F-FDG and ^18^F-BPA PET were $0.65 \pm 0.21$, $0.51 \pm 0.21$, and $0.27 \pm 0.30\;{\text{cm}}$, respectively. In addition, the worse similarities were obtained in the higher metabolic region of MTV50. These results may support the possibility that LAT1 may also be expressed in regions of inadequate glucose metabolism and that high metabolism regions are located in entirely different spatial locations. According to these results, the selection indicators for BNCT should consider the metabolism of BPA.

In assessing heterogeneity within GTV and MTVs using histogram indices for all patients, there were no statistically significant differences in skewness and kurtosis between ^18^F-FDG and ^18^F-BPA PET. However, skewness and kurtosis of MTV40 for RS in ^18^F-BPA PET were significantly higher than those in ^18^F-FDG PET. In general, the accumulation of ^18^F-FDG in various tumor cells is related to the expression of glucose transporter 1 (GRUT1) [27, 28]. The overexpression of hypoxic markers such as hypoxic inducible factor 1α (HIF-1α), hexokinase, carbonic anhydrase 9, and vascular endothelial growth factor seems to play an essential role in its accumulation [27–29]. Since the expression of HIF-1α is regulated by mammalian target of rapamycin (mTOR) [28], the amount of ^18^F-FDG in tumor cells also depends on mTOR signaling. On the other hand, LAT1 provides cancer cells with the essential amino acid not only for protein synthesis but also for stimulating cell growth via mTOR [30, 31]. Since LAT1 is involved in the accumulation of both ^18^F-FDG and ^18^F-BPA agents in cancer cells, it would be reasonable that there is an overlap in the regions of spatial uptake. However, the amount of GRUT1 and LAT1 expression is known to vary by cancer type and stage [19, 23, 32], so it is doubtful that they are entirely matched. It may be rational that there was a divergence between the results of the correlation of the spatial accumulation pattern for all patients and those for each cancer type in our study. In comparison between ^18^F-FDG and ^18^F-BPA, SCC showed a good correlation of SUV_max_, but the ratio of MTVs to GTV was statistically different. RS showed a correlation of both SUV_max_ and SUV_min_, but the ratio of MTV to GTV was not different. However, it was interesting that there was a statistically significant difference in assessing intratumor heterogeneity. In addition to the lack of sufficient sample size for evaluating each cancer type, it would be essential to evaluate the relationship between the distribution of LAT1 and ^18^F-BPA PET in the future. Additionally, to reflect heterogeneous BPA uptake into the clinical outcome, it is vital to develop calculation methods in which the dose distribution reflects the heterogeneous BPA uptake. One of the major options is to use ^18^F-BPA PET information to calculate the heterogeneous BPA uptake and reflect it in dose calculation deriving from treatment planning systems [12, 33]. As a result, it will lead to the future development of BNCT.

There were several limitations in this study. First, the distances of SUV_max_ and the center of mass with MTVs and similarity indices between ^18^F-FDG and ^18^F-BPA PET depend on the registration accuracy between PET and CT images. However, PET and CT images cannot be scanned completely simultaneously, which is still under controversy. Next, two histogram indices, including skewness and kurtosis, were used to evaluate signal heterogeneity within the MTV. Other methods (e.g., texture analysis) may need to be performed to analyze the differences in heterogeneity of its in detail. We focused on three cancer types SCC, Mel, and RS because these cancer types were expected to have some accumulation of ^18^F-BPA for analysis using MTV. Further investigation, including other cancer types, would be necessary. Finally, we compared the spatial accumulation pattern of ^18^F-BPA with ^18^F-FDG, the most widely used PET tracer for diagnosis, to investigate its usefulness for patient selection of BNCT. However, no comparison was made with other amino acid-based radiopharmaceuticals such as ^18^F-FACBC, ^18^F-FET, and ^18^F-FLT, which are likely to show resemble accumulation patterns to ^18^F-BPA. Although there is a possibility that those may be valuable for patient selection in BNCT, the comparison to other amino acid-based tracer need to be discussed carefully, considering the effect on the accuracy of dose calculations in treatment planning for BNCT.

## Conclusions

This study indicated that the spatial parameters between ^18^F-FDG and ^18^F-BPA PET did not correlate, while the non-spatial point parameters did as the results from the previous studies. Due to the limited availability of ^18^F-BPA PET, surrogate indicators using the non-spatial point parameter, such as SUV_max_, derived from ^18^F-FDG PET, have been usually discussed to determine BNCT selection in the previous studies. In comparing ^18^F-FDG and ^18^F-BPA PET in this study, the correlation was indicated not only in SUV_max_ but also in the other non-three-dimensional parameters. However, focusing on the spatial parameters, the similarities in high-accumulation areas, including MTV40 and MTV50, were low. It would be indicated that the high-accumulation region in ^18^F-FDG and ^18^F-BPA was spatially distinct due to the difference in metabolism in each PET tracer. Additionally, SCC, Mel, and RS showed different spatial accumulation patterns in evaluating each cancer type. Therefore, the decision to use ^18^F-FDG PET to determine the indication for BNCT should be more careful compared with using ^18^F-FBPA PET.

## Supplementary Information

**Additional file 1: Fig. S1A.** The correlation of non-spatial point parameters, including SUV_max_, SUV_peak_, SUV_min_, T_max_/N, and T_min_/N between ^18^F-FDG and ^18^F-BPA PET for squamous cell carcinoma patients. T_max_/N; maximum tumor-to-normal tissue count ratio, T_min_/N minimum tumor-to-normal tissue count ratio.

**Additional file 2: Fig. S1B.** The correlation of non-spatial point parameters, including SUV_max_, SUV_peak_, SUV_min_, T_max_/N, and T_min_/N between ^18^F-FDG and ^18^F-BPA PET for melanoma patients. T_max_/N; maximum tumor-to-normal tissue count ratio, T_min_/N minimum tumor-to-normal tissue count ratio.

**Additional file 3: Fig. S1C.** The correlation of non-spatial point parameters, including SUV_max_, SUV_peak_, SUV_min_, T_max_/N, and T_min_/N between ^18^F-FDG and ^18^F-BPA PET for rhabdomyosarcoma patients. T_max_/N; maximum tumor-to-normal tissue count ratio, T_min_/N minimum tumor-to-normal tissue count ratio.

**Additional file 4: Fig. S2A.** The correlation of TLA in GTV, MTV40, and MTV50 between ^18^F-FDG and ^18^F-BPA PET for squamous cell carcinoma. TLA; total lesion activity.

**Additional file 5: Fig. S2B.** The correlation of TLA in GTV, MTV40, and MTV50 between ^18^F-FDG and ^18^F-BPA PET for melanoma. TLA; total lesion activity.

**Additional file 6: Fig. S2C.** The correlation of TLA in GTV, MTV40, and MTV50 between ^18^F-FDG and ^18^F-BPA PET for rhabdomyosarcoma patients. TLA; total lesion activity.

**Additional file 7: Fig. S3.** The volume ratio of MTV40 and MTV50 to GTV in ^18^F-FDG and ^18^F-BPA PET for squamous cell carcinoma, melanoma, and Rhabdomyosarcoma.

**Additional file 8: Fig. S4A.** The similarity indices, including Dice similarity coefficient, Jaccard coefficient, and Mean distance to agreement, of MTV40 and MTV50 between ^18^F-FDG and ^18^F-BPA PET for squamous cell carcinoma.

**Additional file 9: Fig. S4B.** The similarity indices, including Dice similarity coefficient, Jaccard coefficient, and Mean distance to agreement, of MTV40 and MTV50 between ^18^F-FDG and ^18^F-BPA PET for melanoma.

**Additional file 10: Fig. S4C.** The similarity indices, including Dice similarity coefficient, Jaccard coefficient, and Mean distance to agreement, of MTV40 and MTV50 between ^18^F-FDG and ^18^F-BPA PET for Rhabdomyosarcoma.

**Additional file 11: Fig. S5A.** The histogram indices, including skewness and kurtosis, of GTV, MTV40, and MTV50 in ^18^F-FDG and ^18^F-BPA PET for squamous cell carcinoma.

**Additional file 12: Fig. S5B.** The histogram indices, including skewness and kurtosis, of GTV, MTV40, and MTV50 in ^18^F-FDG and ^18^F-BPA PET for melanoma.

**Additional file 13: Fig. S5C.** The histogram indices, including skewness and kurtosis, of GTV, MTV40, and MTV50 in ^18^F-FDG and ^18^F-BPA PET for rhabdomyosarcoma.

## Data Availability

The datasets generated and/or analyzed during the current study are available from the corresponding author upon reasonable request.

## Abbreviations

BPA: Para-boronophenylalanine
BNCT: Boron neutron capture therapy
CR: Complete response
CT: Computed tomography
DSC: Dice similarity coefficient
FDG: Fluorodeoxyglucose
GTV: Gloss tumor volume
JC: Jaccard coefficient
LAT1: L-type amino acid transporter 1
MDA: Mean distance to agreement
Mel: Melanoma
mTOR: Mammalian target of rapamycin
MTV: Metabolic tumor volume
PET: Positron emission tomography
RS: Rhabdomyosarcoma
SCC: Squamous cell carcinoma
SUV: Standardized uptake value
*T*/*N*: Tumor-to-normal tissue ratio
