# Supplementary figures and images for: Analyzing spatial distribution between 18F-fluorodeoxyglucose and 18F-boronophenylalanine positron emission tomography to investigate selection indicators for boron neutron capture therapy
Source: EJNMMI Phys. 2022 Dec 19;9:89. doi: 10.1186/s40658-022-00514-7 (PMC9763526; doi:10.1186/s40658-022-00514-7)

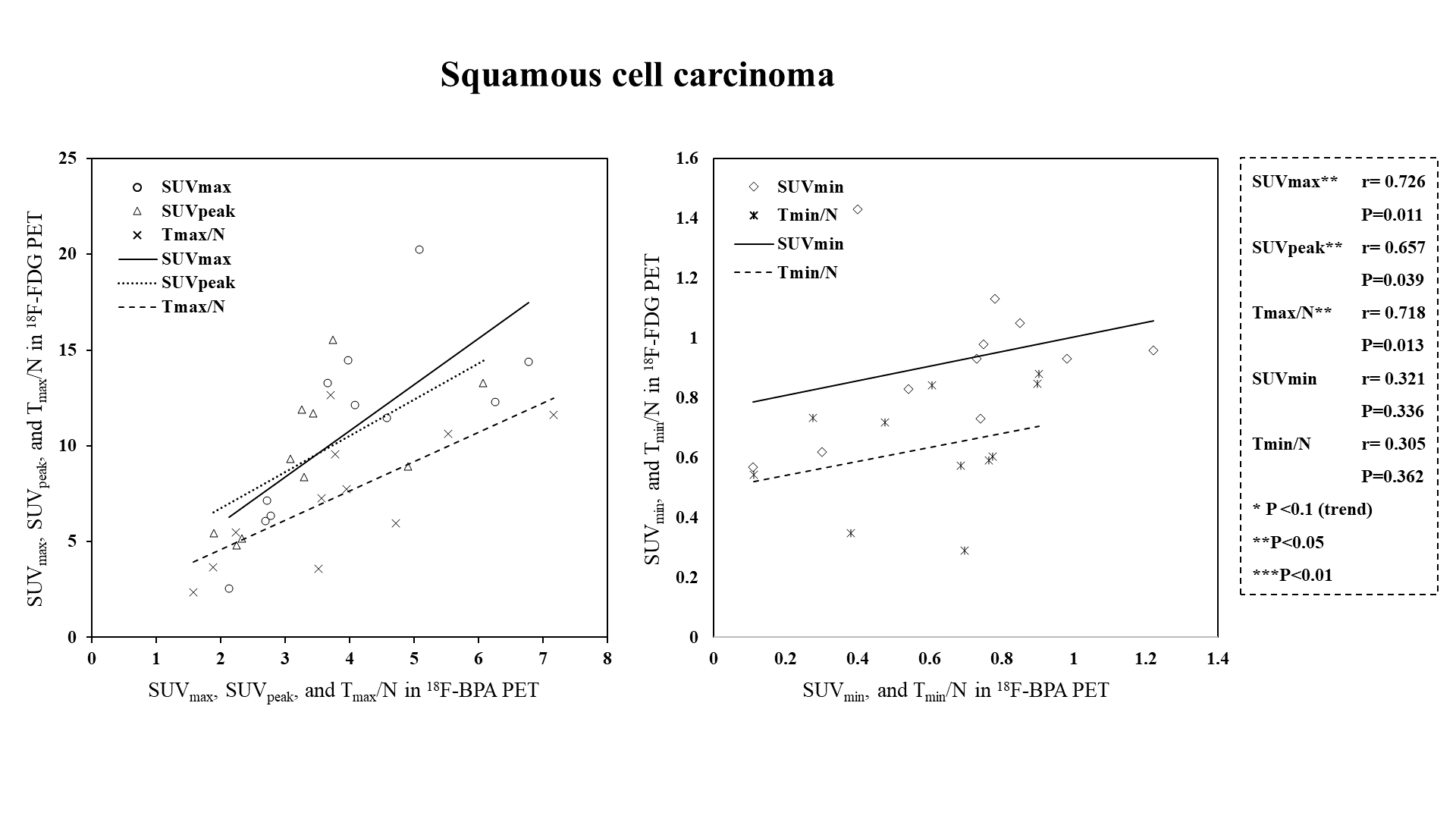

Supplement: Supplementary file 1 — Additional file 1: Fig. S1A. The correlation of non-spatial point parameters, including SUVmax, SUVpeak, SUVmin, Tmax/N, and Tmin/N between 18F-FDG and 18F-BPA PET for squamous cell carcinoma patients. Tmax/N; maximum tumor-to-normal tissue count ratio, Tmin/N minimum tumor-to-normal tissue count ratio. [file 40658_2022_514_MOESM1_ESM.png]

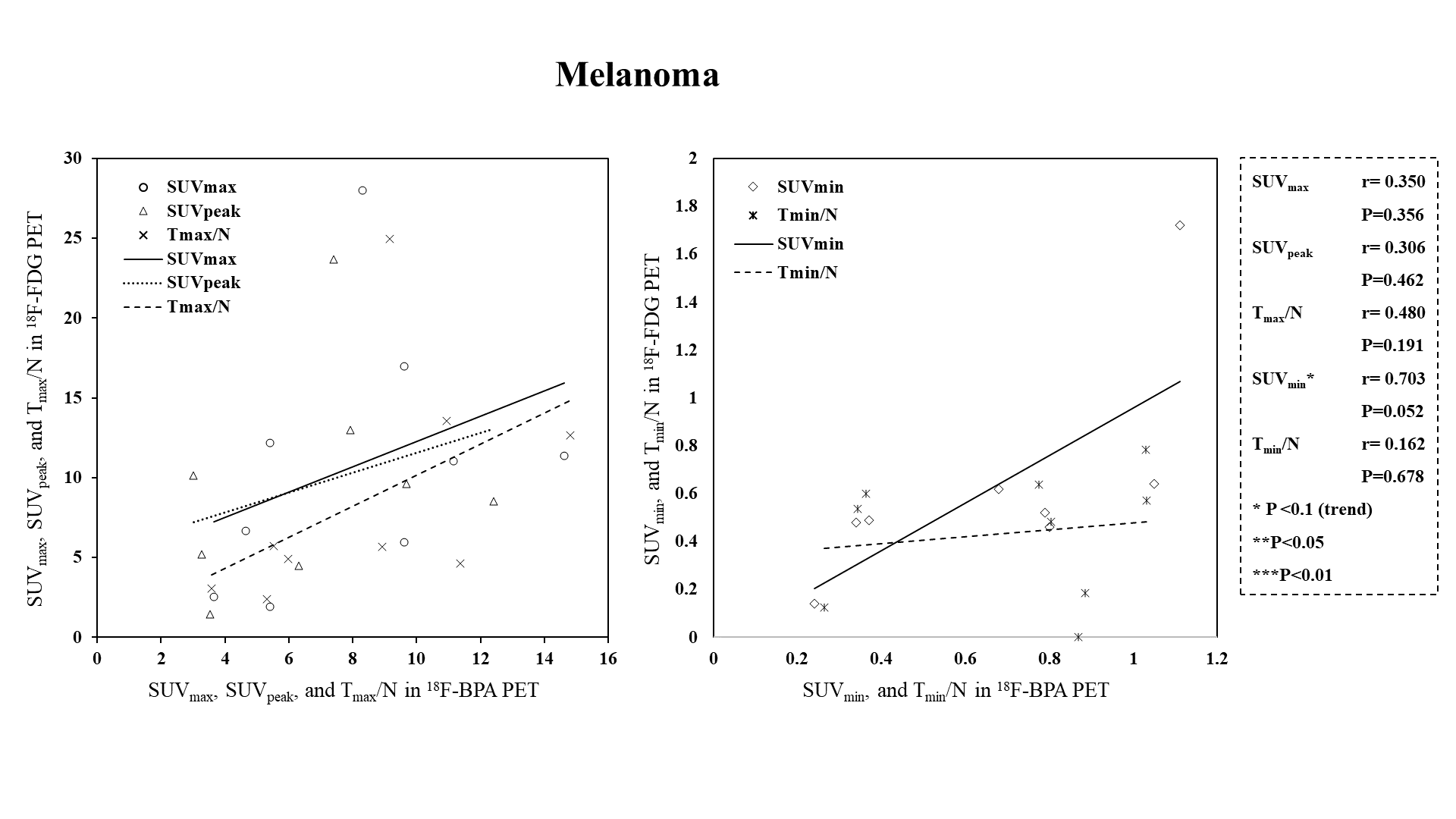

Supplement: Supplementary file 2 — Additional file 2: Fig. S1B. The correlation of non-spatial point parameters, including SUVmax, SUVpeak, SUVmin, Tmax/N, and Tmin/N between 18F-FDG and 18F-BPA PET for melanoma patients. Tmax/N; maximum tumor-to-normal tissue count ratio, Tmin/N minimum tumor-to-normal tissue count ratio. [file 40658_2022_514_MOESM2_ESM.png]

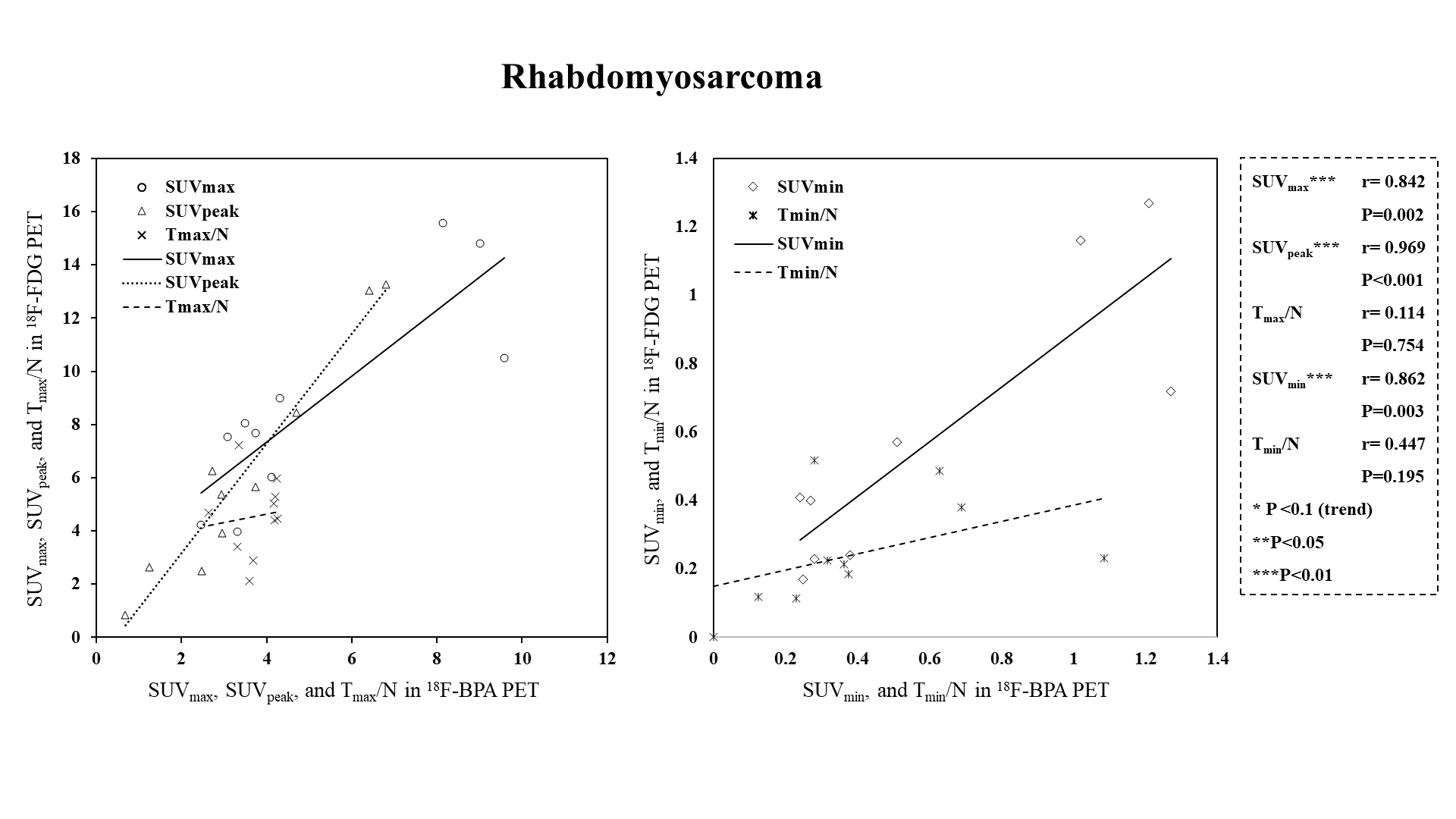

Supplement: Supplementary file 3 — Additional file 3: Fig. S1C. The correlation of non-spatial point parameters, including SUVmax, SUVpeak, SUVmin, Tmax/N, and Tmin/N between 18F-FDG and 18F-BPA PET for rhabdomyosarcoma patients. Tmax/N; maximum tumor-to-normal tissue count ratio, Tmin/N minimum tumor-to-normal tissue count ratio. [file 40658_2022_514_MOESM3_ESM.png]

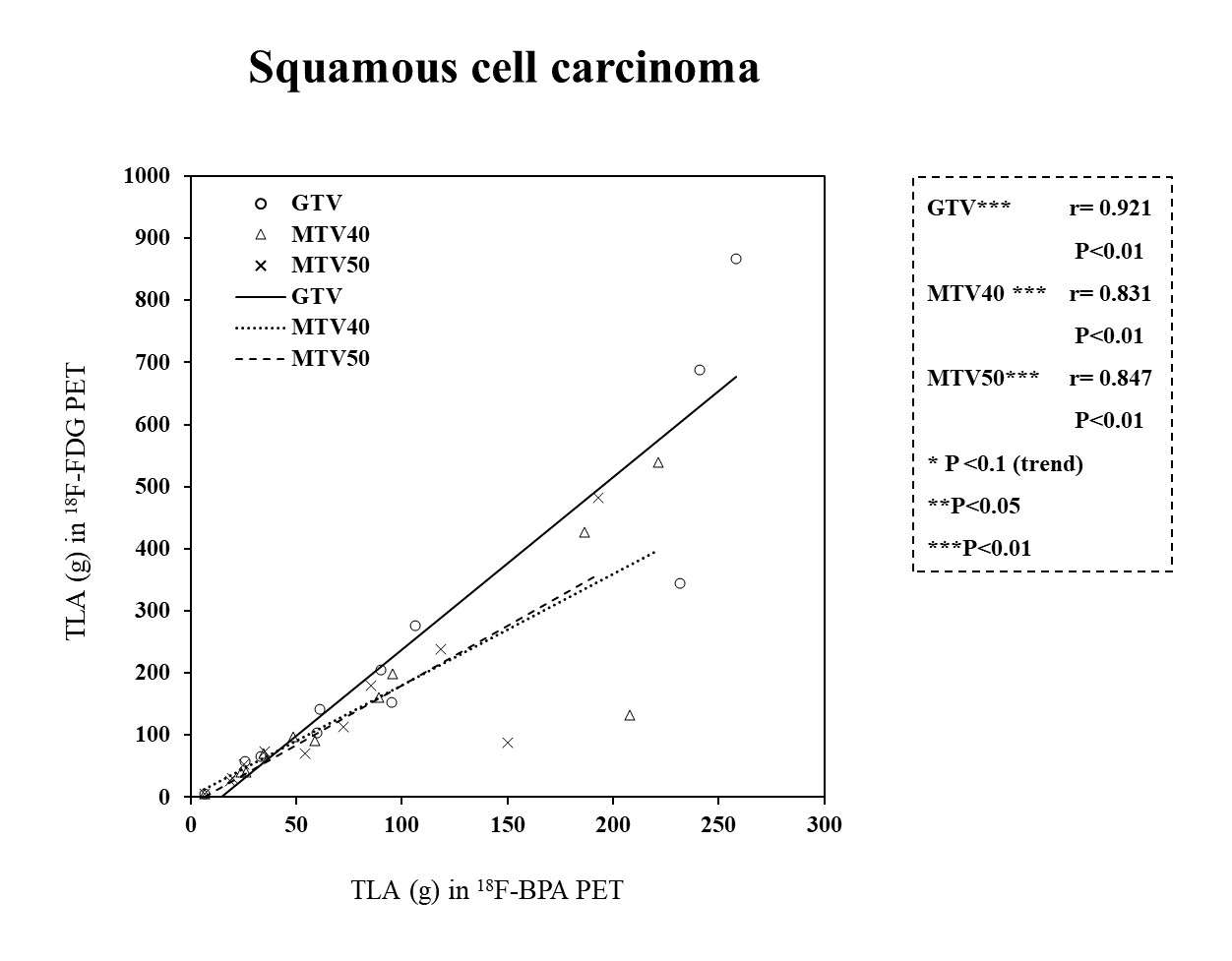

Supplement: Supplementary file 4 — Additional file 4: Fig. S2A. The correlation of TLA in GTV, MTV40, and MTV50 between 18F-FDG and 18F-BPA PET for squamous cell carcinoma. TLA; total lesion activity. [file 40658_2022_514_MOESM4_ESM.jpg]

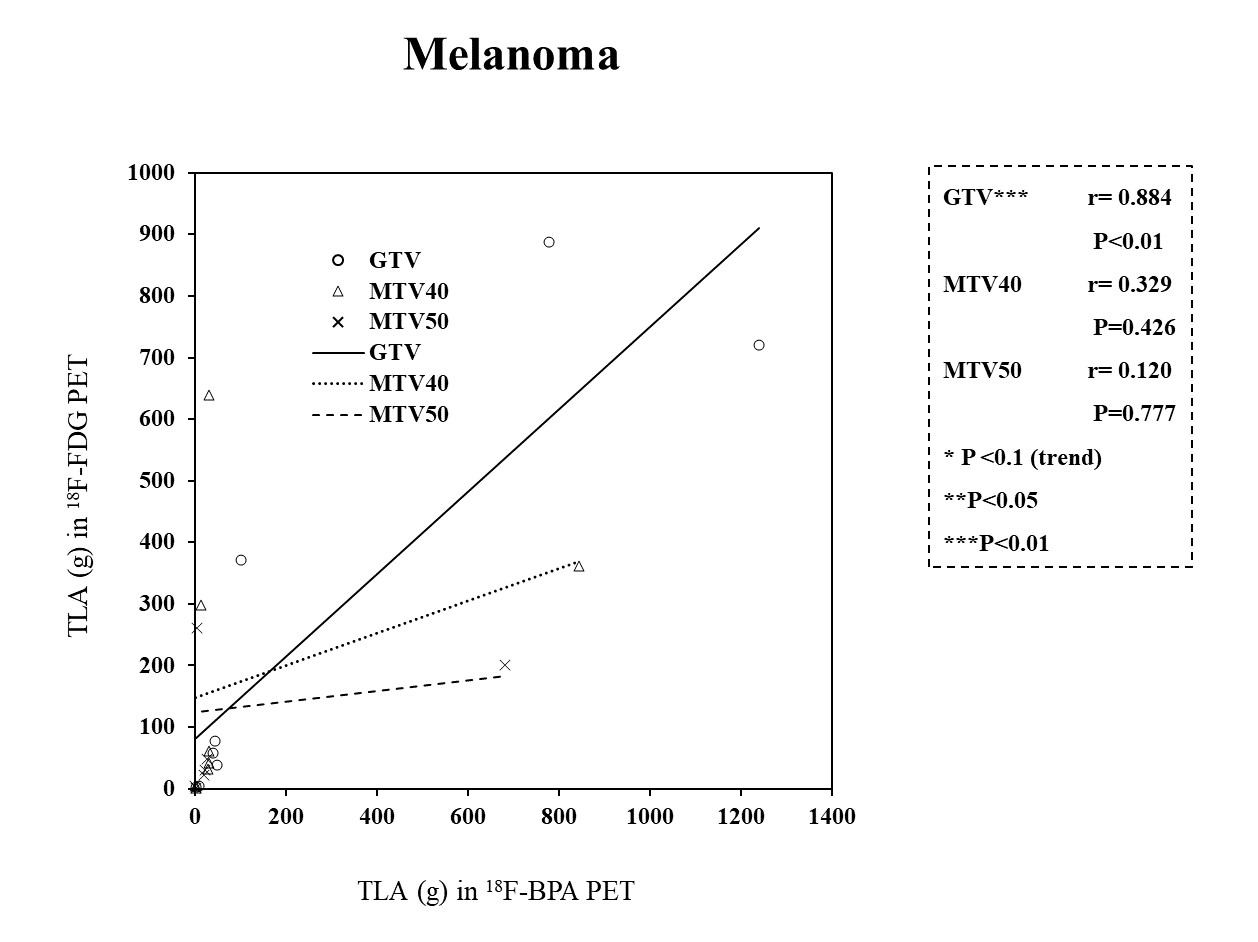

Supplement: Supplementary file 5 — Additional file 5: Fig. S2B. The correlation of TLA in GTV, MTV40, and MTV50 between 18F-FDG and 18F-BPA PET for melanoma. TLA; total lesion activity. [file 40658_2022_514_MOESM5_ESM.jpg]

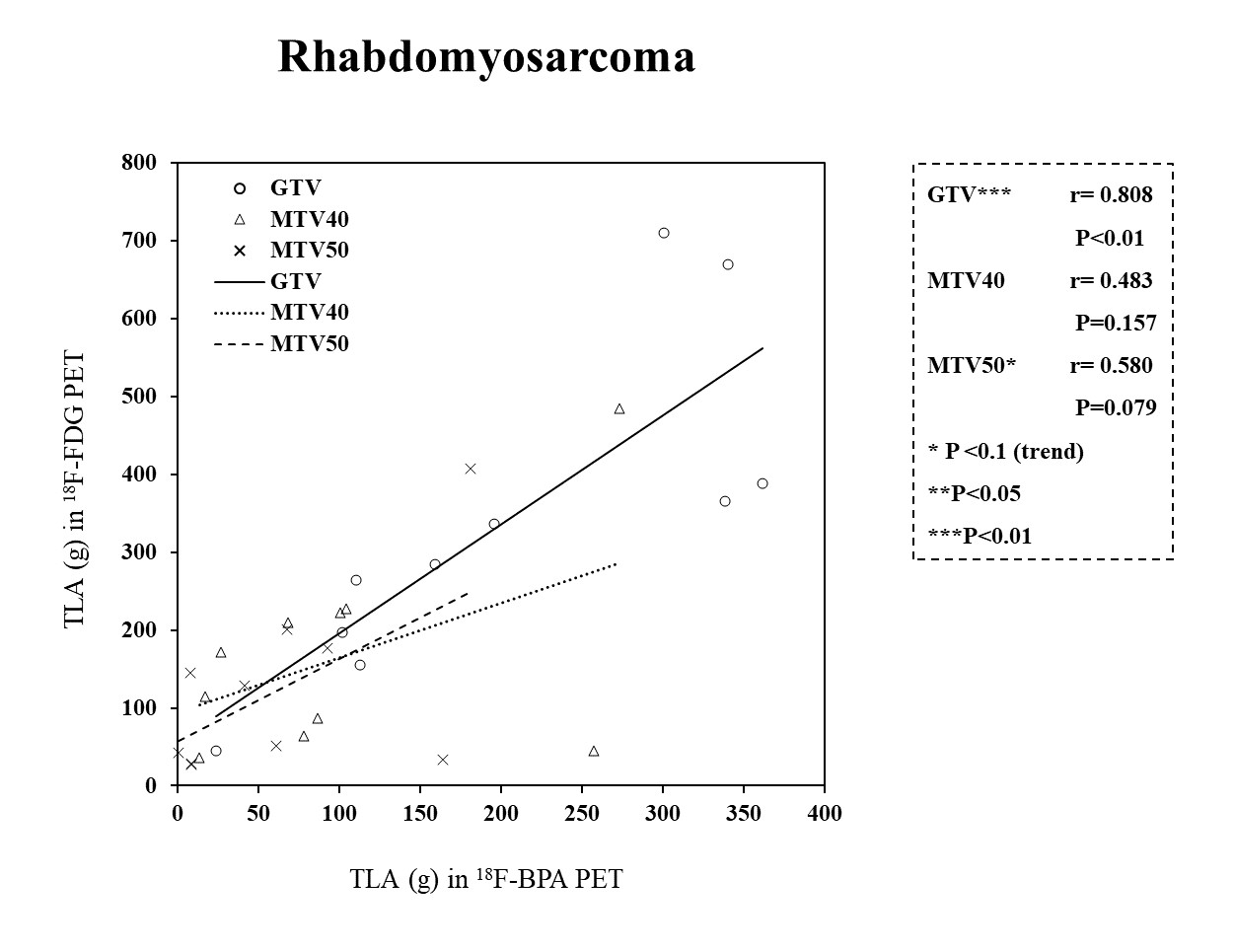

Supplement: Supplementary file 6 — Additional file 6: Fig. S2C. The correlation of TLA in GTV, MTV40, and MTV50 between 18F-FDG and 18F-BPA PET for rhabdomyosarcoma patients. TLA; total lesion activity. [file 40658_2022_514_MOESM6_ESM.jpg]

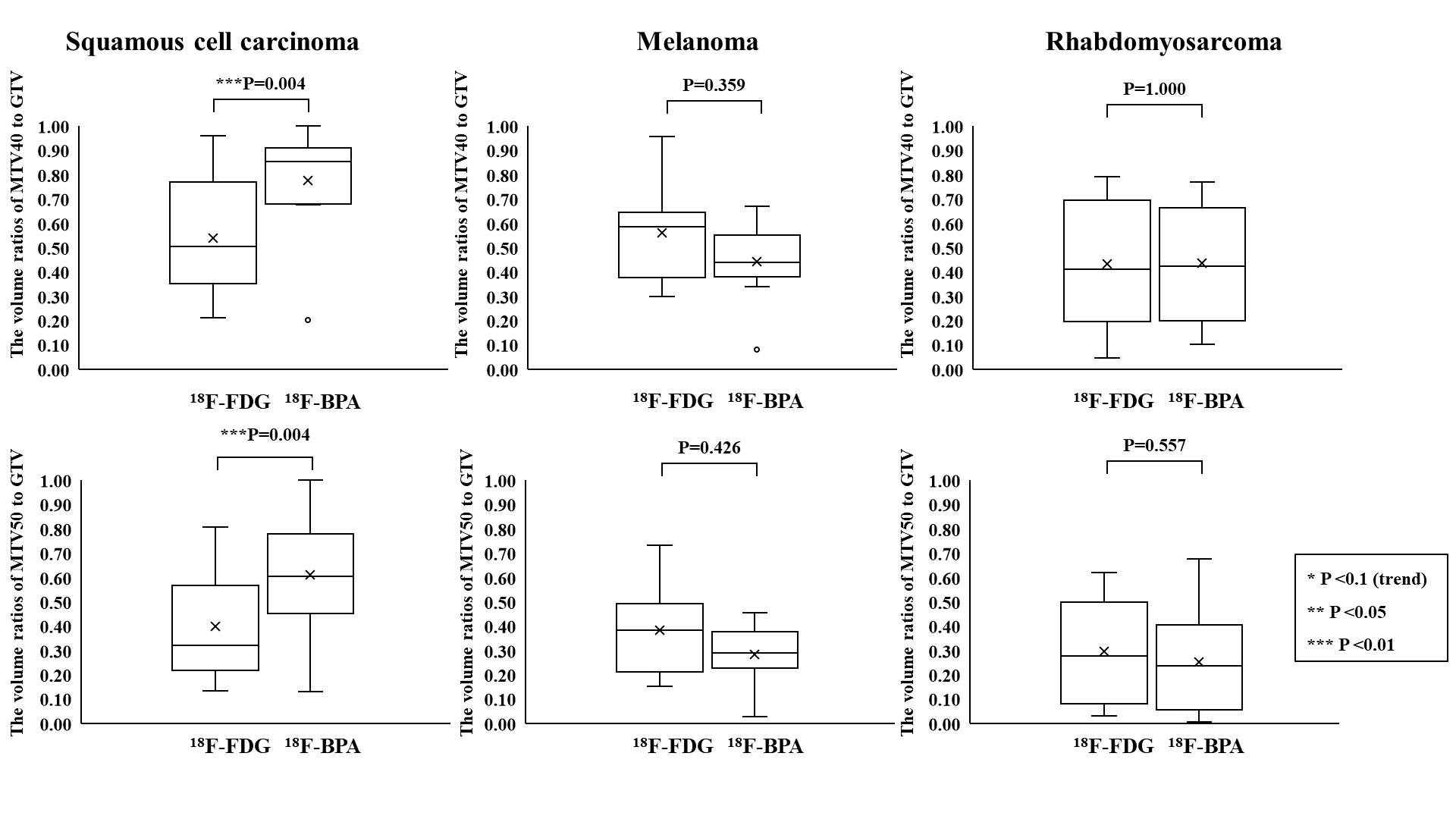

Supplement: Supplementary file 7 — Additional file 7: Fig. S3. The volume ratio of MTV40 and MTV50 to GTV in 18F-FDG and 18F-BPA PET for squamous cell carcinoma, melanoma, and Rhabdomyosarcoma. [file 40658_2022_514_MOESM7_ESM.png]

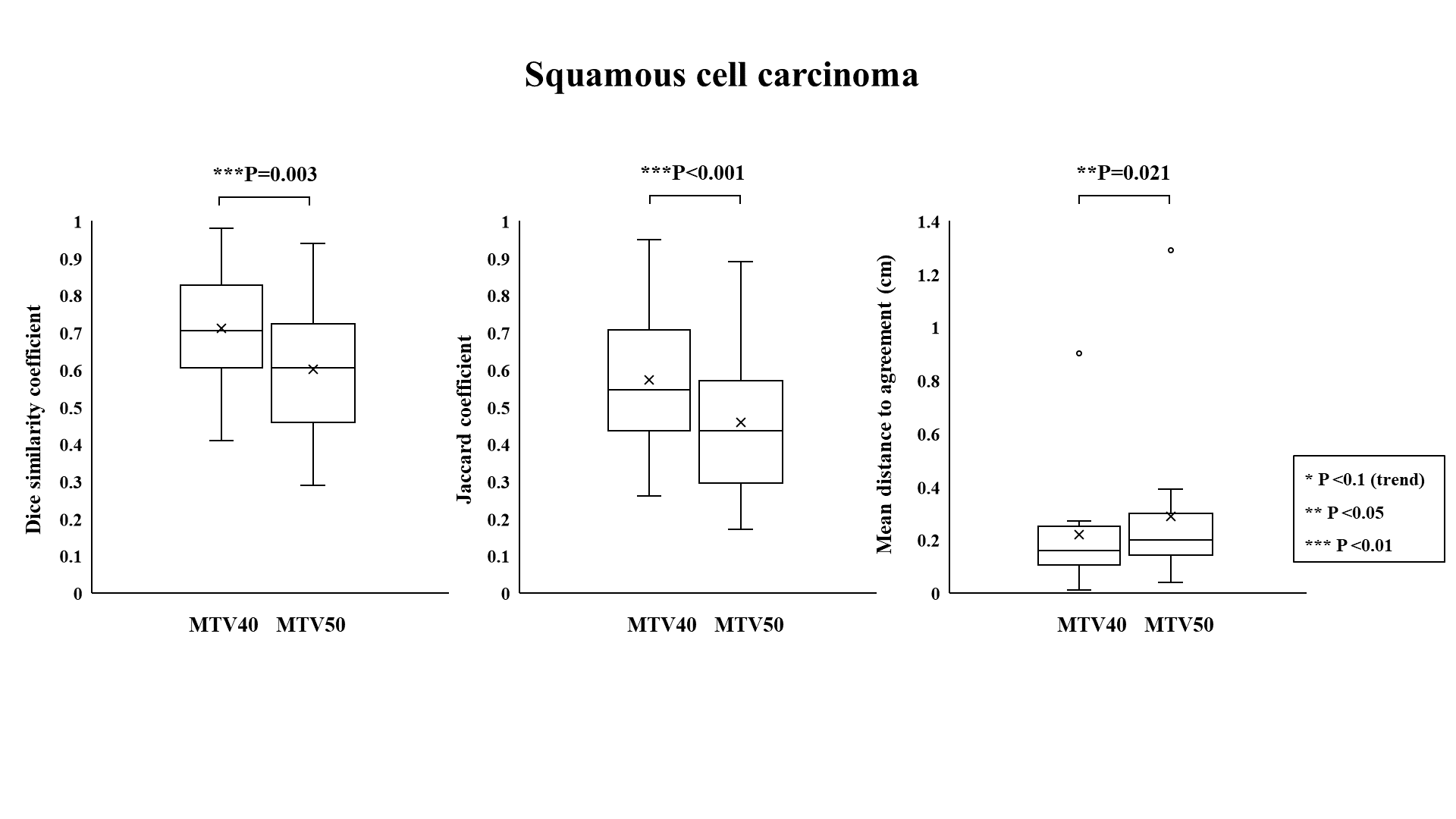

Supplement: Supplementary file 8 — Additional file 8: Fig. S4A. The similarity indices, including Dice similarity coefficient, Jaccard coefficient, and Mean distance to agreement, of MTV40 and MTV50 between 18F-FDG and 18F-BPA PET for squamous cell carcinoma. [file 40658_2022_514_MOESM8_ESM.png]

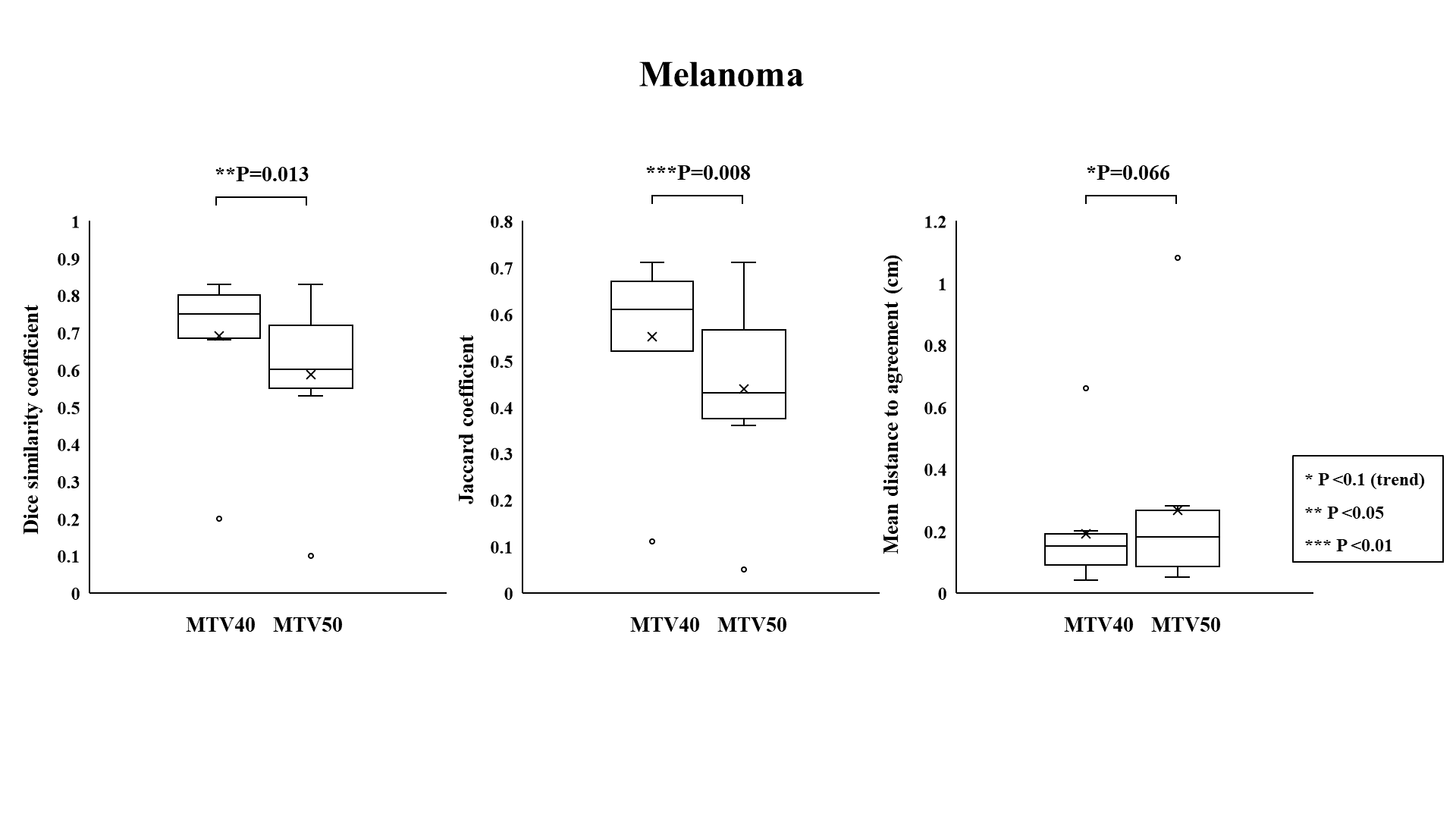

Supplement: Supplementary file 9 — Additional file 9: Fig. S4B. The similarity indices, including Dice similarity coefficient, Jaccard coefficient, and Mean distance to agreement, of MTV40 and MTV50 between 18F-FDG and 18F-BPA PET for melanoma. [file 40658_2022_514_MOESM9_ESM.png]

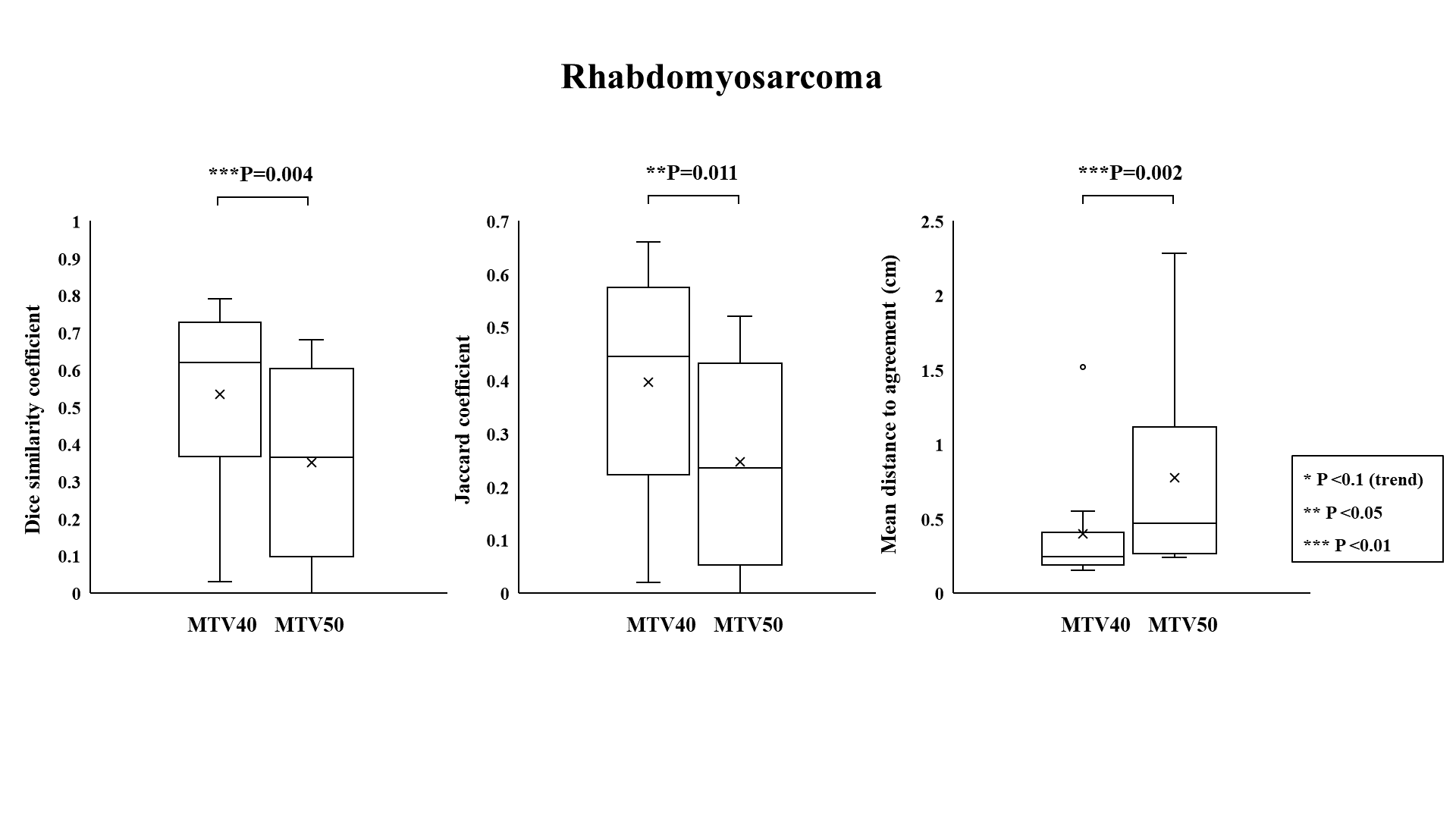

Supplement: Supplementary file 10 — Additional file 10: Fig. S4C. The similarity indices, including Dice similarity coefficient, Jaccard coefficient, and Mean distance to agreement, of MTV40 and MTV50 between 18F-FDG and 18F-BPA PET for Rhabdomyosarcoma. [file 40658_2022_514_MOESM10_ESM.png]

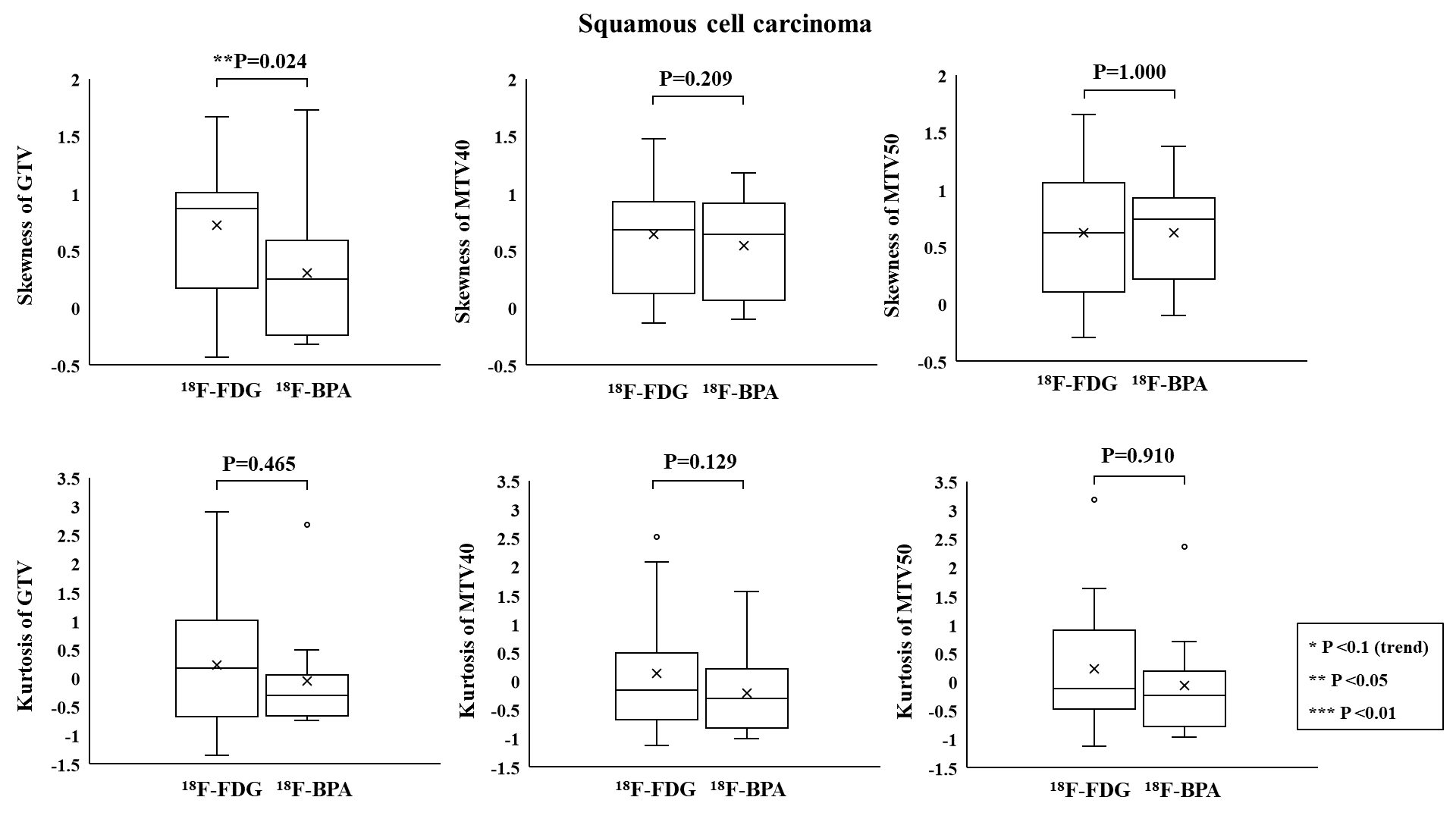

Supplement: Supplementary file 11 — Additional file 11: Fig. S5A. The histogram indices, including skewness and kurtosis, of GTV, MTV40, and MTV50 in 18F-FDG and 18F-BPA PET for squamous cell carcinoma. [file 40658_2022_514_MOESM11_ESM.png]

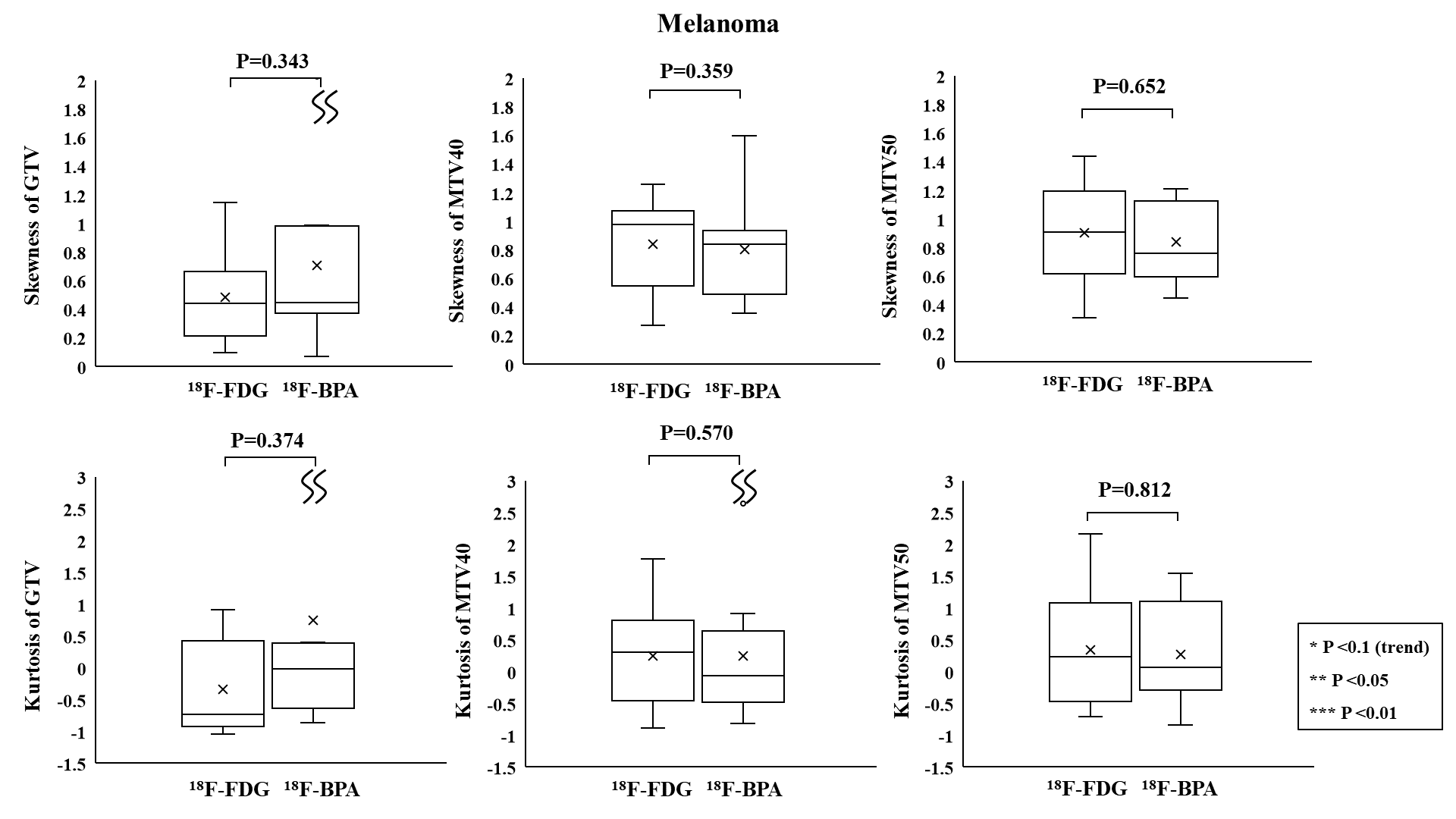

Supplement: Supplementary file 12 — Additional file 12: Fig. S5B. The histogram indices, including skewness and kurtosis, of GTV, MTV40, and MTV50 in 18F-FDG and 18F-BPA PET for melanoma. [file 40658_2022_514_MOESM12_ESM.png]

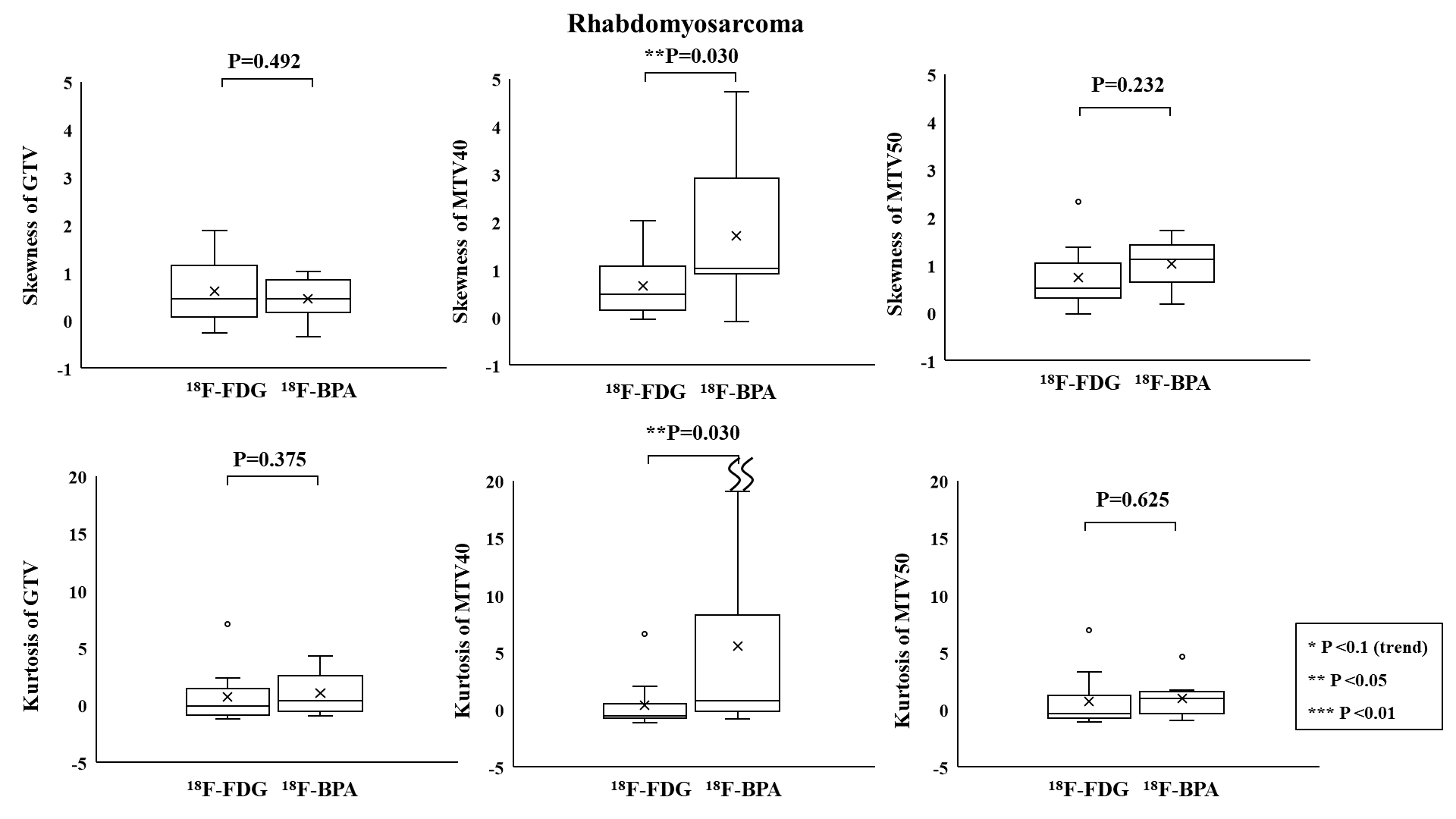

Supplement: Supplementary file 13 — Additional file 13: Fig. S5C. The histogram indices, including skewness and kurtosis, of GTV, MTV40, and MTV50 in 18F-FDG and 18F-BPA PET for rhabdomyosarcoma. [file 40658_2022_514_MOESM13_ESM.png]
